# Mechanisms of Herbal Nephroprotection in diabetes mellitus

**DOI:** 10.1155/2020/5710513

**Published:** 2020-06-30

**Authors:** Dorin Dragoș, Maria Mirabela Manea, Delia Timofte, Dorin Ionescu

**Affiliations:** ^1^Faculty of General Medicine, “Carol Davila” University of Medicine and Pharmacy, str. Dionisie Lupu nr. 37, sect 1, Bucharest 020021, Romania; ^2^Nephrology Clinic of University Emergency Hospital, Splaiul Independentei nr. 169, sect. 5, Bucharest 050098, Romania; ^3^National Institute of Neurology and Cerebrovascular Diseases, Şos. Berceni, Nr. 10-12, Sector 4, Bucharest 041914, Romania; ^4^Dialysis Department of University Emergency Hospital, Splaiul Independentei nr. 169, sect. 5, Bucharest 050098, Romania

## Abstract

Diabetic nephropathy (DN) is a leading cause of kidney morbidity. Despite the multilayered complexity of the mechanisms involved in the pathogenesis of DN, the conventional treatment is limited to just a few drug classes fraught with the risk of adverse events, including the progression of renal dysfunction. Phytoceuticals offer a promising alternative as they act on the many-sidedness of DN pathophysiology, multitargeting its intricacies. This paper offers a review of the mechanisms underlying the protective action of these phytoagents, including boosting the antioxidant capabilities, suppression of inflammation, averting the proliferative and sclerosing/fibrosing events. The pathogenesis of DN is viewed as a continuum going from the original offense, high glucose, through the noxious products it generates (advanced glycation end-products, products of oxidative and nitrosative stress) and the signaling chains consequently brought into action, to the harmful mediators of inflammation, sclerosis, and proliferation that eventually lead to DN, despite the countervailing attempts of the protective mechanisms. Special attention was given to the various pathways involved, pointing out the ability of the phytoagents to hinder the deleterious ones (especially those leading to, driven by, or associated with TGF-*β* activation, SREBP, Smad, MAPK, PKC, NF-*κ*B, NLRP3 inflammasome, and caspase), to promote the protective ones (PPAR-*α*, PPAR-*γ*, EP4/Gs/AC/cAMP, Nrf2, AMPK, and SIRT1), and to favorably modulate those with potentially dual effect (PI3K/Akt). Many phytomedicines have emerged as potentially useful out of *in vitro* and *in vivo* studies, but the scarcity of human trials seriously undermines their usage in the current clinical practice—an issue that stringently needs to be addressed.

## 1. Introduction

**Figure 1:**
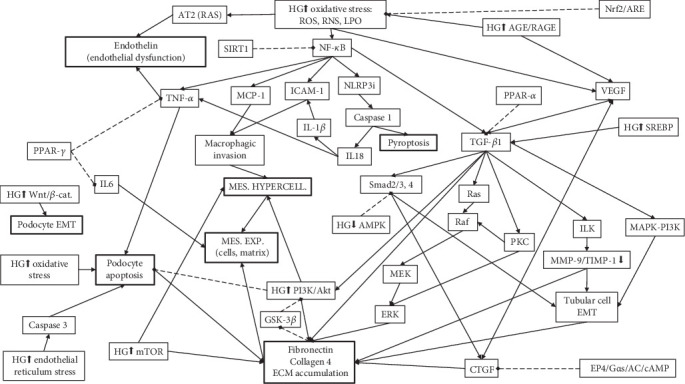
Pathways and mediators of DN relevant for the action mechanisms of the phytoagents active in DN. The schematic is meant to highlight the complexity of the factors and interconnections involved in DN pathogenesis, although it leaves out many of them lest it might become unintelligible. HG↑: activated/increased/induced by high glucose (HG); HG↓: inactivated/decreased by HG, mes.: mesangial; exp.: expansion; hypercell.: hypercellularity; continuous arrowhead-ended lines indicate a stimulating effect; dotted diamond-ended lines indicate an inhibitory effect.

In most countries, diabetic nephropathy (DN) (also known as diabetic kidney disease) is the main cause of chronic kidney disease (CKD) [1]. DN results from the interplay of several distinct but highly interconnected high glucose- (HG-) induced pathways set into motion by aggressive factors, such as oxidative stress [2] and advanced glycation end-products (AGEs), which trigger signaling chains that generate mediators able to instigate reactive processes, including inflammation, cellular proliferation, and interstitial matrix expansion [3]. Oxidative stress and inflammation enhance each other, resulting in a vicious circle leading to glomerular sclerosis and interstitial fibrosis [4].  Figure 1 illustrates some of the most important mechanisms of DN, although it is by no means exhaustive.

Among the *inflammatory* mediators involved in DN are nuclear factor kappa-B (NF-*κ*B), monocyte chemotactic protein- (MCP-) 1, and intercellular adhesion molecule- (ICAM-) 1. Attracted and activated by MCP-1 and helped by ICAM-1 (promoted by NF-*κ*B), circulating monocytes invade the kidney [5]. Together with resident mesangial cells proliferation, this leads to the *mesangial hypercellularity* characteristic for the diabetic glomerulus [6]. *Glomerulosclerosis* is the hallmark of DN [7] and consists in proteins of extracellular matrix (ECM) (mostly collagen types I, III, and IV and fibronectin [8]) gradually and inexorably encumbering the mesangium, either by lumping together in nodular lesions or by diffusely invading and expanding the interstitial space separating the glomerular loops [9]. The ECM proteins accumulation is the result of excessive production (mostly by mesangial cells) coupled with insufficient proteolysis by mesangial matrix metalloproteinases (MMPs) [10]. However, the pathological deposition of ECM proteins is not limited to the glomerular interstitium but also involves the tubular interstitium and the glomerular basement membrane, explaining its increased thickness [8]. The cellular component of both glomerulus and tubulointerstitium is altered by epithelial-mesenchymal transdifferentiation (EMT) [4]. *Growth-promoting pathways* involved in DN are those driven by p38 mitogen-activated protein kinase (p38MAPK) [11], mammalian target of rapamycin (mTOR), and phosphatidylinositol 3-kinase (PI3K)/Akt/glycogen synthase kinase- (GSK-) 3*β*, the last two sometimes acting in concert [12]. It is not clear whether PI3K/Akt activation should be considered protective or deleterious [13]. *Endothelial dysfunction* [14] and *endoplasmic reticulum* (ER) *stress* are other important links in the pathophysiological chain leading to DN [15, 16].

Among the pharmaceuticals used for slowing the progression of DN are lipid-lowering agents and renin-angiotensin-aldosterone system blockers, especially angiotensin-converting enzyme inhibitors and angiotensin receptor blockers [17], but also mineralocorticoid receptor antagonists [18]. However, the use of these drugs is fraught with the risk of adverse events, including liver and muscle injury, progression of renal dysfunction, and hyperkalemia. Consequently, therapeutic alternatives devoid of such adverse effects are needed. Worldwide herbs have been used for the treatment of diabetes [19], including in the two most practiced systems of traditional medicine: ayurvedic [20] and traditional Chinese medicine [21]. Due to the complexity of their composition, medicinal herbs have, over the modern antidiabetic drugs, the advantage of influencing multiple pathogenic mechanisms [22, 23] and being devoid of significant adverse effects [24]. Herbal treatment can complement and even increase the efficiency of the conventional one, the synergistic effect of the combined treatment allowing for dose reduction and delaying the need for insulin therapy [23]. The aim of this article is to review the mechanisms underlying the protective action of these phytomedicines.

## 2. Material and Method

A PubMed (https://www.ncbi.nlm.nih.gov/pubmed) search for (“diabetic nephropathy”[Title/Abstract] OR “diabetic kidney disease”[Title/Abstract]) AND (plant[Title/Abstract] OR herbal[Title/Abstract]) and a ScienceDirect (https://www.sciencedirect.com/) search for (“diabetic nephropathy” OR “diabetic kidney disease”) AND (plant OR herbal) provided the articles employed in this review. The authors endeavored to include (almost) all the relevant papers, preferring those attempting to define the molecular/cellular background of the renoprotective action and those investigating clearly defined active compounds or herbal products (articles on herbal formulas with unclear components were left out). The results of this search are summarized in Table 1.

We considered that a study proved that a herbal product is actually nephroprotective if it demonstrated lower levels of glomerular injury markers (in most cases, albuminuria and/or proteinuria) or improved kidney histology or function (stated as such or reflected by lower serum levels of urea and/or creatinine) in the subjects who took the herbal product. We considered the herbal products for which neither of these effects was demonstrated as only potentially nephroprotective. Practically all the *in vivo* studies (there was only one exception), done on either human or animal subjects (including those that also had an *in vitro* component), proved the ability of the investigated phytoceuticals to protect the kidney, while none of the exclusively *in vitro* studies did so. Consequently, for each mechanism or pathway, the data were separated into *in vitro*, *in vivo*, and clinical, pointing out that the *in vitro* studies suggest potentially protective mechanisms, while the *in vivo* ones demonstrate actually protective mechanisms.

In most of the *in vivo* studies, nephroprotection consisted in the ability of the phytoceuticals to lessen the degree of glomerular and/or tubulointerstitial injury at the completion of the study, which may be equated with the ability to delay the progression of DN. No study could demonstrate the complete prevention of DN; therefore, none of the plant products was able to prevent the onset of DN.

In order to assess the strength of the evidence in the area of the clinical trials regarding the efficiency of herbals in DN, a secondary search was performed: on PubMed for (“diabetic nephropathy”[Title/Abstract] OR “diabetic kidney disease”[Title/Abstract]) AND (plant[Title/Abstract] OR herbal[Title/Abstract]) AND (trial[Title/Abstract]) and on ScienceDirect for (“diabetic nephropathy” OR “diabetic kidney disease”) AND (plant OR herbal) AND (trial). The Jadad scale was employed for evaluating the quality of the human trials [25]. The trials with Jadad score of 1, 2, 3, 4, and 5 (designated as J1, J2, J3, J4, and J5, respectively) were considered of low, low-to-moderate, moderate, moderate-to-high, and high quality, respectively.

## 3. Herbal Nephroprotection

### 3.1. Herbal Products Decreasing Serum Glucose Level and Peripheral Resistance

As HG is the point of depart in the pathophysiological chain leading to DN, optimal glycemic control prevents CKD or at least delays its onset and slows its pace [26]. Insulin resistance may be involved in the genesis of DN [27].

#### 3.1.1. *In Vivo* Studies—Actually Nephroprotective Mechanisms

Many of the investigated herbal products are able to decrease glucose level (*Allium sativum* [28], *Artemisia sieberi* [29], *Bacopa monnieri* [30], *Hypericum perforatum* [31], *Punica granatum* [32], and *Terminalia chebula* [33] to name only a few—see Table 2 for a complete list) and some of them are also able to decrease insulin resistance (*Cladophora glomerata* [34], *Panax notoginseng* [35], Huangqi decoction [36] etc.—see Table 2) which corroborates or explains their glucose-lowering effect. This is evident especially on the animal models conceived to mimic as good as possible the actual human disease, such as high-fat diet/low-dose streptozotocin type 2 diabetic Wistar albino rats in which some of the most important features of human type 2 diabetes are recognizable: hyperglycemia, insulin resistance, heightened oxidative stress, and structural and functional kidney deterioration. In this animal model, obesity-related insulin resistance, and not lack of insulin, is the cause of altered glucose metabolism. Insulin resistance (that may be attenuated by ellagic acid) is the result of the inflammatory response triggered by adipose tissue infiltration by immune cells spurred by proinflammatory cytokines such as interleukin- (IL-) 1*β*, IL-6, and tumor necrosis factor- (TNF-) *α* produced by NF-*κ*B-activated adipocytes [37].

### 3.2. Herbal Products Decreasing Oxidative Stress and AGEs Production

*Oxidative stress* is the consequence of antioxidant protective mechanisms being overwhelmed by the reactive oxygen species (ROS) generation. It leads to reactive nitrogen species (such as peroxynitrite) production, lipid peroxidation, inflammatory pathways activation [via NF-*κ*B, protein kinase C (PKC), etc.], apoptosis, and mesangium (both cells and matrix) expansion [4, 38]. Acting by means of RAGE (receptor for AGEs), *AGEs* induce inflammation, oxidative stress, apoptosis, and exuberant ECM protein synthesis [39], one of the mediators being ROS production [8].

#### 3.2.1. *In Vitro* Studies—Potentially Nephroprotective Mechanisms

Paeoniflorin and oxypaeoniflora (from *Paeonia suffruticosa*), improved the protection against AGE-induced inflammatory and oxidative damage by boosting glutathione peroxidase and catalase activities, hampering both the cellular (macrophage migration), and humoral (transcription factors, cytokines) components of inflammation [40]. Curcumin and demethoxycurcumin (from *Curcuma longa*) prevented AGE-induced apoptosis of mesangial cells [41].

Oxidative stress also activates the inflammatory response and undermines cell viability. Consequently, moringa isothiocyanate (from *Moringa oleifera*), able to activate nuclear factor erythroid-derived 2 (*Nrf2*), the chief regulator of the antioxidant response, also suppresses transforming growth factor-*β*1 (TGF-*β*1) signaling and the production of proinflammatory cytokines by macrophages [42]. Silybin (from *Silybum marianum*) preserves the viability of HG-injured podocytes by decreasing superoxide production, reduced nicotinamide-adenine dinucleotide phosphate (NADPH) oxidase activity, and NAPDH oxidase 4 (NOX4) expression [43].

The *PI3K/Akt* pathway increases insulin sensitivity, averts apoptosis, and is important for recovery after kidney injury, but it may also deleteriously promote cell proliferation and fibrogenesis in DN. A similar action may have *mTOR* pathway, and these two pathways may act in concert. Inactivating phosphorylation of GSK-3*β* may be one of the mechanisms mediating the proliferative and fibrogenetic effects of PI3K/Akt activation [12]. Berberine (from *Berberis vulgaris*) induces PI3K/Akt signaling pathway, which results in the activation of Nrf2 (and its target genes, including heme oxygenase-1) and of hypoxia-inducible factor 1*α*. Both these factors reduce HG-induced apoptosis, particularly when associated with oxidative [44] and hypoxic injury [45], respectively.

#### 3.2.2. *In Vivo* Studies—Actually Nephroprotective Mechanisms

Many studies have pointed out the *antioxidant* properties of the investigated herbal products (catechin [46], *Cornus officinalis* [47], dihydroquercetin [48], etc.—see Table 2), translated in their ability to *diminish the burden of ROS* (quercetin [49], etc.), *reactive nitrogen species* (tocotrienol [50], etc.), and *lipid peroxidation* products (breviscapine [51, 52], *Flammulina velutipes* [53], *Hibiscus sabdariffa* [54], and many more—see Table 2). Beside decreasing malondialdehyde, *Artemisia campestris* also lowered the level of nitric oxide (NO) and advanced oxidation protein products [55]. Most of these plant-derived products are also able to *increase the antioxidant capacity* (*Anogeissus acuminata* [56], *Pleurotus eryngii* [57], *Punica granatum* [32], etc.—see Table 2 for a complete list).

Suppressing the oxidative stress prevents inflammation and cell death. Therefore, the antioxidant effect maintains cells alive (as silybin does with the HG-damaged podocytes [43]) and decreases inflammation (garlic reduced not only the burden of oxidative stress but also the level of TNF-*α* [28]). The antioxidant armamentarium of some phytoagents includes the activation of *Nrf2*, in conjunction with ameliorating mitochondrial dysfunction, dampening the inflammatory response, and bolstering the function of antioxidant enzymes (curcumin [58]) or with suppressing TGF/Smad signaling and the glomerular accumulation of fibronectin and collagen 4 (*Hydrangea paniculata* [59]). Puerarin (from *Pueraria lobata*) prevents podocyte foot process effacement and boosts the expression of *podocyte* slit diaphragm proteins such as nephrin and podocin, beside attenuating oxidative and nitrosative stress, and one of their consequences, the activation of MMP-9 [60].

Apart from the antioxidant capacity, some herbal products have also demonstrated an ability to *decrease the production of AGEs* (*Bacopa monnieri* [30], coconut water [61], diosgenin [62], *Paeonia emodi* [63], *Physalis angulata* [64], etc.—see Table 2). A cyclitol from soybean, d-pinitol, has been shown to decrease both AGEs and inflammation-promoting factors [2].

#### 3.2.3. Human Studies—Actually Nephroprotective Mechanisms

A study done on human subjects has proved the ability of silymarin to boost the antiproteinuric activity of renin-angiotensin system (RAS) inhibitors [65].

### 3.3. Herbal Products with Anti-Inflammatory Activity

*Inflammation* is a key pathophysiologic component in the genesis of DN [66–68]. The involvement of microinflammation in the pathogenesis of DN justifies the interest for anti-inflammatory herbal products in preventing DN—this concept may be correlated with the thousands years old employment of “heat-clearing” herbs for the treatment of DN in Traditional Chinese Medicine [69]. Herbal products may decrease inflammation by multiple ways, such as lowering the level of proinflammatory cytokines (TNF-*α*, IL-1*β*, IL-6, IL-12, etc.), decreasing the factors promoting inflammatory cells infiltration (chemokines such as MCP-1 and adhesion molecules including ICAM-1 and vascular cell adhesion molecule-1), and modulating the inflammatory pathways and/or the activity of transcription factors.

#### 3.3.1. *In Vitro* Studies—Potentially Nephroprotective Mechanisms

Purple corn (*Zea mays*) anthocyanins hindered the interaction between NF-*κ*B and canonical TGF-*β*1 (i.e., *Smad*) pathways. Consequently, it thwarted ECM expansion by both promoting ECM degradation and reducing new ECM formation as a result of TGF-*β*1 signaling inhibition blocking the induction of ICAM-1 and MCP-1 (responsible for connective tissue growth factor expression) and the secretion of collagen 4 (essential for mesangial hyperplasia). ECM dissolution was the consequence of higher membrane type-1 MMP and lower tissue inhibitor of MMP- (TIMP-) 2 expression [70].

*AMPK* (5′ adenosine monophosphate-activated protein kinase) acts as an energy sensor and undermines TGF-*β*1/Smad pathway by hindering Smad4 translocation into the nucleus, thereby impeding ECM accumulation [71]. *Coreopsis tinctoria* and its main component, the chalconoid marein, blocked both TGF-*β*1/Smad (by means of p-AMPK) and NF-*κ*B pathways, and consequently attenuated inflammation, mesangial cell proliferation, and fibrogenesis [72].

Crocin, the carotenoid from *Crocus sativus* responsible for its saffron color, has also been proven a podocyte-friendly substance. It is able to foster the integrity of glomerular filtration barrier, demonstrated by higher levels of slit diaphragms markers: nephrin, podocin, and CD2-associated protein. Moreover, it diminishes oxidative stress and the proinflammatory response of the podocytes (by NF-*κ*B inactivation) [73].

*SIRT1* (Sirtuin 1) suppresses the activity of NF-*κ*B by deacetylating its RelA/p65 subunit [74, 75]. Puerarin from the roots of *Pueraria lobata* increased the level and activity of *SIRT1* protein in podocytes, followed by enhanced SIRT1-mediated deacetylation (hence, inactivation) of NF-*κ*B and reduced NOX4 expression [76].

#### 3.3.2. *In Vivo* Studies—Actually Nephroprotective Mechanisms

Downregulating the activity of *NF-κB* dependent pathways is one of the most explored anti-inflammatory renoprotective mechanism of the herbal products. In most studies, NF-*κ*B inactivation is accompanied by lower levels of proinflammatory factors, including various combinations of proinflammatory cytokines: IL-1 (and IL-1 receptor), IL-6, interferon-*γ*, and TNF-*α* (*Morus alba* [77]), TNF-*α*, IL-1*β*, and IL-6 (*Paederia foetida* [78], d-pinitol from soybean [2]).

Other studies demonstrated, aside from *NF-κB* inactivation and lower levels of proinflammatory *cytokines*, a decrease in the expression of *chemokines* (mainly MCP-1) and of the factors spurring sclerosis/fibrosis (especially TGF*-β1*), thereby warding off macrophage infiltration (curcumin [79]) and suppressing the production of collagen 4 and fibronectin, and hence ECM expansion and glomerulosclerosis (ellagic acid [37], Shen-Yan-Fang-Shuai Formula [80]). Added to these effects, the inhibition *cyclooxygenase-2* and *inducible NO synthase* boosted the anti-inflammatory potency of green tea polyphenols [81], of *Hypericum perforatum* [31], and of curcumin analogue B06 (in the latter case probably by means of blocking the *JNK* (c-Jun N-terminal kinase)*/NF-κB* signaling [66]). Suppressing the production of *vasoactive* factors (vascular endothelial growth factor, endothelin-1) resulted in an all-encompassing protective spectrum for North American ginseng, which has been shown to decrease inflammation, fibrosis, and mesangial expansion, to improve the oxidative and metabolic status [82], and to suppress AGEs generation [83]. Other members of the Panax genus have also been shown to prevent diabetic glomerular lesions (*P. notoginseng* [35]). The aptitude to block both *inflammation* and *fibrosis* is also manifested by plant extracts that conjointly inhibit NF-*κ*B and canonical TGF-*β*1 (i.e. *Smad*) pathways (*Prunella vulgaris* [84]), berberine [85]). Arctigenin from *Fructus arctii* also can improve the viability and function of the podocytes conjointly with attenuating the NF-*κ*B-mediated inflammatory effects due to protein phosphatase 2 A-mediated decrease in p65 NF-*κ*B activating phosphorylation [86].

Several herbal products hampered the production of *ROS* conjointly to inhibiting *NF-κB* dependent inflammatory pathways (puerarin [76]) and the production/activation of various proinflammatory *cytokines* (*Paederia foetida* [78]), adhesion molecules, and fibrosis promoting cytokines (isorhamnetin, present in onions, sea buckthorn, and various other medicinal plants [87]). Secoisolariciresinol diglucoside (from *Linum usitatissimum*) is able not only to diminish the inflammatory and oxidative aggression on the kidney cells, but also to prevent their death, as reflected by higher levels of *antiapoptotic* markers survivin and B-cell lymphoma-2 (Bcl-2) [88].

*Hypericum perforatum* seems to exert an all-embracing nephroprotective action, the anti-inflammatory and antioxidant effects being complemented by antifibrosing and antiapoptotic activity (inhibition of caspase-3 and cytochrome c), and enhanced expression of *PPAR* (peroxisome proliferator-activated receptor)*-γ* [31]. Strawberry (*Fragaria × ananassa*) extracts also combine the PPAR-*γ* pathway activating effect with an anti-inflammatory one, associated with suppression of several fatty acid synthesis genes and of the sterol regulatory element-binding protein (*SREBP*) transcription factor [89], one of the inducers of TGF-*β*1 that may be activated by a HG-milieu [90]. The inactivation of both *SREBP* and NF-*κ*B pathways, coupled with antioxidant activity and decreased production of AGEs, was proven for the fruit of *Cornus officinalis*, having morroniside, loganin, and 7-O-galloyl-D-sedoheptulose as the main active compounds [47].

The *NLRP3* (nucleotide binding and oligomerization domain-like receptor family pyrin domain-containing 3) inflammasome triggers inflammatory events by means of activating caspase-1, which leads to both proinflammatory cytokines (such as IL-1*β* and IL-18) activation and to pyroptosis, a type of inflammatory cell death [91]. The dihydroflavone dihydroquercetin blocked the activation of NLRP3 inflammasome, besides reducing cell proliferation, ROS generation, and the expression of renal fibrosis-associated proteins [48].

The activation of the *GSK-3β* pathway is known to ameliorate diabetes-induced kidney injury. GSK-3*β* inactivation by *PI3K/Akt*-mediated phosphorylation increases protein synthesis associated with diabetic glomerular hypertrophy and sclerosis [12, 92]. Akt (protein kinase B) is involved in metabolism (particularly glucose metabolism), growth, proliferation, and survival/apoptosis. PI3K is a key regulator of the multi-step process controlling Akt activation [93]. Emodin, the main active component of rhubarb (*Rheum officinale*), exerted its anti-inflammatory, antiapoptotic [decrease in B-cell lymphoma 2-associated X protein (Bax) and caspase-3 expression], and antioxidative activities by triggering *PI3K/Akt/GSK-3β* signaling pathway [94]. However, it is presently not clear whether PI3K/Akt induction should be considered protective or deleterious for the diabetic kidney, as among the phytoagents useful for preventing DN some turn this pathway on, while others turn it off [13].

#### 3.3.3. Human Studies—Actually Nephroprotective Mechanisms

In one of the very few human trials, berberine lowered the level of vascular cell adhesion molecule-1 and C-reactive protein, as well as the urinary markers of kidney injury. It also favorably tipped the oxidative stress balance (decreasing lipid peroxidation and nucleic acid oxidation and augmenting the total-antioxidant capacity) and improved renal hemodynamics. Hence, berberine may emerge as a nephroprotective agent able to complement standard hypotensive and hypoglycemic treatment [95]. In another trial on human subjects, *Dioscorea bulbifera* provided a better control than fosinopril of blood pressure, metabolic, biologic, and inflammatory parameters in patients with DN [96].

### 3.4. Preventing Endothelial Dysfunction

The nephroprotective effect of some herbal products includes the prevention of endothelial dysfunction, an attribute of DN epitomized by the imbalance between endothelium-derived vasodilators and vasoconstrictors, the former preventing thrombosis and proliferation and the latter promoting atheroma formation [14]. Endothelial dysfunction is reflected by biomarkers such as endothelin 1 [97], homocysteine [98], and IL-6 (a proinflammatory cytokine) [99]. Induced by angiotensin II [100], oxidative stress, inflammation, and hypoxia [46], endothelin 1 promotes afferent and efferent arterioloconstriction with declining glomerular filtration rate [101].

#### 3.4.1. *In Vivo* Studies—Actually Nephroprotective Mechanisms

A component of green tea, (+)-catechin, has been proven to mitigate endothelial dysfunction, as reflected by diminished endothelin 1 levels, beside exerting antifibrosing and antioxidant activity [46].

#### 3.4.2. Human Studies—Actually Nephroprotective Mechanisms

A study on stable diabetic CKD patients revealed the ability of *Salacia chinensis* to forestall endothelial dysfunction, demonstrated by a decrease in the levels of homocysteine and IL-6 ([102]).

### 3.5. Preventing ER stress

*Endoplasmic reticulum* (ER) *stress* is an important link in the pathophysiological chain leading to DN, consisting in improperly folded proteins piling up in the ER lumen and consequently unchaining the unfolded protein response (UPR), which may lead to either cell protection or cell death by apoptosis [15, 16]. SERCA dysfunction is an important inductor of ER stress [103].

#### 3.5.1. *In Vivo* Studies—Actually Nephroprotective Mechanisms

*Abelmoschus manihot* extract (Huangkui capsule) attenuates ER stress in correlation with reduced JNK activation [104]. Astragaloside IV (derived from *Astragalus membranaceus*) dampens ER stress and, consequently, podocyte apoptosis [15] by means of SERCA2 [103].

### 3.6. Herbal Products with Antifibrosing/Antiproliferative Activity

TGF*-β*1 has a crucial role in the progression of DN [105] leading to EMT [106], ECM expansion, collagen biosynthesis, and renal cell growth [10]. The result is glomerular and whole kidney hypertrophy [107] that, together with thickening of the glomerular basement membrane, are emblematic for DN [108].

#### 3.6.1. *In Vitro* Studies—Potentially Nephroprotective Mechanisms

Paeoniflorin, pentagalloylglucose, and paeonol from the root bark of *Paeonia suffruticosa* have been shown to suppress TGF-*β*1 signaling, thereby reducing the levels of fibronectin ([109]). Purple corn anthocyanins too interfere with TGF-*β*1/Smad pathway (and NF-*κ*B signaling), beside reducing the expression of connective tissue growth factor and collagen 4 and accelerating ECM dissolution [70].

#### 3.6.2. *In Vivo* Studies—Potentially Nephroprotective Mechanisms

A study performed on spontaneously hypertensive rats with streptozotocin-induced diabetes has shown that activating phosphorylation of AMPK by cocoa enriched with polyphenols may suppress *TGF-β1/Smad* pathway (by blocking Smad2 phosphorylation), resulting in diminished levels of TGF-*β*1, collagen 4, and fibronectin ([110]).

#### 3.6.3. *In Vivo* Studies—Actually Nephroprotective Mechanisms

Tea catechins, a class of flavonoids, have been shown to decrease interstitial fibrosis [3]. *Coccinia indica* could prevent the accumulation of ECM by decreasing fibronectin and laminin in association with antioxidant activity [111].

Arguably the most efficient mechanism for preventing fibrosis/sclerosis is downregulation of its key promoter, *TGF-β1*, and of the related signaling pathways. Activation of TGF-*β*1 type I receptors leads to Smad2 and Smad3 phosphorylation, followed by binding to Smad4, the resultant oligomeric complex translocating into the nucleus, where it regulates the transcription of key proteins responsible for renal fibrogenesis [112]. Abating TGF-*β*1 expression is typically associated with lower levels of fibronectin (rhein [113]), collagen (*Cordyceps militaris* [114]), or both, sometimes accompanied by antiapoptotic effects, as is the case for *Psoralea corylifolia* seed extract and two of its major components, isopsoralen and psoralen. These phytoagents downregulated proapoptotic proteins [such as cleaved poly (ADP-ribose) polymerase and Bcl-2-associated death promoter (Bad)] and buttressed cell viability, as indicated by higher levels of survival markers p-Bad (ser112) and B-cell lymphoma- (Bcl-) 2 [115].

Declining levels of TGF-*β*1, collagen 4, and fibronectin may be correlated with *TGF-β1/Smad* pathway mitigation (Tangke decoction [116], *Hydrangea paniculata* [59]) by means of activating phosphorylation of AMPK leading to reduced phosphorylation of Smad2 via diminished NOX4 expression (berberine [117]). The shrinking levels of TGF-*β*1 may also be coupled with reduced activity and expression of *PKC*, associated with antioxidant activity (breviscapine [52]) and anti-inflammatory effect (reduced macrophage infiltration) (again breviscapine [51]), and with suppression of AGEs/RAGE/PKC-*β*/TGF-*β*1 signaling pathway (berberine [118]). The decrease in TGF-*β*1 level may also be associated with inhibition of the *RAS* and of the myofibroblast proliferation (low *α*-smooth muscle actin) (Qidan Dihuang decoction [119]) or with anti-inflammatory action reflected in lower levels of inflammatory promoters (*Paeonia lactiflora* [120]).

Modulation of *G protein/AC* (adenylyl cyclase)/*cAMP* (cyclic adenosine monophosphate) signaling pathway by means of altering G protein-coupled receptor kinases may be connected with dwindling TGF-*β*1 and collagen 4 expression in the case of berberine [121]. Berberine also interfered with *prostaglandin E_2_/E prostanoid receptor (EP) 1/Gαq/Ca^2+^* pathway, its depressing effects being associated with a decline in mesangial cells proliferation [122]. By blocking the *SREBP*, which is known to upregulate TGF-*β*1 [90], curcumin was linked to diminished expression of TGF-*β*1, EMT suppression (diminished vimentin), and podocyte protection (low desmin and high synaptopodin and connexin 43) [108]. Tangshen Formula combines the antifibrosing effect with an autophagy-inducing one, achieved by inhibiting the pathway leading from promyelocytic leukemia zinc finger protein (*PLZF*) activation to a decline in autophagy and autophagy-induced collagen 3 dissolution. Hence, the effect of Tangshen Formula is more autophagy and less collagen 3 accumulation, and consequently less tubulointerstitial ECM deposition [123].

#### 3.6.4. Human Studies—Actually Nephroprotective Mechanisms

A multicenter randomized, double-blind, placebo-controlled trial on the efficacy of Tangshen Formula in DN yielded promising results: after a mere 6 months, the improvements in proteinuria and estimated glomerular filtration rate (eGFR) were significant [124], as was the decrease in liver-type fatty acid binding protein [125], a biomarker correlated with the severity and prognosis of DN [126]. Longer duration interventions are clearly needed for defining the role of this formula in preventing DN.

### 3.7. Herbal Products Blocking Epithelial-Mesenchymal Transdifferentiation

Fibrosis and ECM synthesis are bolstered by EMT, in which the epithelial cells trade their epithelial features (reflected by proteins such as E-cadherin) for mesenchymal ones (correlated with the expression of *α*-smooth muscle actin and vimentin, among others) [4]. TGF-*β*1 is one of the main inducers of EMT by means of both the canonical pathway (TGF-*β*/Smad2/3) and the non-canonical one (MAPK/PI3K) [106]. Consequently, all herbal products able to forestall TGF-*β*1 generation are potentially apt to thwart EMT.

#### 3.7.1. *In Vitro* Studies—Potentially Nephroprotective Mechanisms

Matrix metalloproteinase- (MMP-) 9 promotes EMT by degrading type IV collagen of the basement membranes (thereby facilitating the migration of the transdifferentiated epithelial cells through the altered basement membrane) and by preventing the dissolution of ECM, which furthers renal interstitial fibrosis. The overactivity of MMP-9 results from insufficient inhibition by TIMP-1. The MMP-9/TIMP-1 imbalance is induced by integrin-linked kinase (ILK), a downstream mediator on a pathway driven by TGF-*β*1 [127]. Rhein hampers EMT by suppressing ILK, thus correcting the excessively high MMP-9/TIMP-1 [127].

#### 3.7.2. *In Vivo* Studies—Actually Nephroprotective Mechanisms

Caveolin-1 is involved in regulating cell junctions, including E-cadherin/*β*-catenin complex, an adhesion complex linked to actin filaments. The *β*-catenin component functions as a coactivator in the Wnt/*β*-catenin signaling pathway that accelerates the EMT of podocytes [128–130]. *Curcumin* inhibits caveolin-1 Tyr(14) phosphorylation which stabilizes caveolin-1 and *β*-catenin, thus blocking EMT of podocytes [130]; it also suppresses the EMT of tubular cells, as reflected by lower levels of vimentin, an important marker of this process [108].

### 3.8. Herbal Products that Restore Autophagy

By degrading damaged cellular components, autophagy, an adaptive response of cells exposed to various environmental stresses, is essential for maintaining the structural and functional well-being of podocytes [103, 131, 132]. Consequently, its malfunction in DN leads to podocyte loss and proteinuria [133]. As it acts as a protective mechanism [134], its inactivation contributes to disease progression [135]. Nonetheless, some authors have found autophagy activation in DN—this seems to be an early, short-lived event, followed by autophagy depression in the long run [135].

#### 3.8.1. *In Vitro* Studies—Potentially Nephroprotective Mechanisms

Several pathways converge in regulating autophagy and are the points of impact of the various herbal products that enhance cell viability by activating autophagy: AMPK (berberine [136]), mTOR (hispidulin [137]), PI3K/AKT (curcumin [132]), and *β*-arrestin-1 (tripterygium glycosides [138]). Besides increasing autophagy, celastrol also dampens inflammation, oxidative stress, and apoptosis and hence increases podocyte viability, as reflected by enhanced expression of nephrin [139]. Still, there are herbal products whose favorable action is associated with reduced autophagy, such as astilbin, a flavanonol found in several plants, including *Hypericum perforatum*. The beneficial effect of astilbin on HG-treated human proximal tubular epithelial cells was associated with diminished apoptosis and autophagy, resulting from Akt induction (mirrored by higher levels of p-Akt) [140]. This may be explained by autophagy activation early in the course of DN.

#### 3.8.2. *In Vivo* Studies—Actually Nephroprotective Mechanisms

*In vivo* (or combined *in vivo* and *in vitro*) studies confirmed the ability of herbal products to act on the pathways already mentioned: AMPK (astragaloside IV [103]), mTOR (triptolide [141]), and AMPK/mTOR (triterpenic acids-enriched fraction of *Cyclocarya paliurus* [142], mangiferin [139]). Several other pathways have also been revealed: SIRT1 (*Abelmoschus manihot* [143], astragaloside IV [131], resveratrol [144]), MAPKs (ferulic acid [145]), PLZF (Tangshen Formula [123]), and miR-18a-5p/atactic telangiectasis mutation (resveratrol [133]). Some herbal products have the ability to act on the pathways driven by one or another environmental stressors, such as oxidative stress (*Vigna angularis* [146]) or hypoxic stress (resveratrol [144]).

### 3.9. Herbal Products Apt to Activate Protective Pathways

The mechanisms able to delay the progression of DN include, besides autophagy, the pathways driven by PPARs [147], EP4/Gs (protein Gs)/AC/cAMP [148], Nrf2 [42, 58, 149], AMPK [71], adiponectin [150], and SIRT1 [74, 75].

#### 3.9.1. *In Vitro* Studies—Potentially Nephroprotective Mechanisms

Activated by environmental stressors, p38MAPK stimulates cellular growth, differentiation, and apoptosis [151, 152]. Activation of p38MAPK signaling is associated with the development of DN, being involved especially in the progression of interstitial fibrosis [11]. Emodin (from *Rheum palmatum*) interferes with p38MAPK pathway, thereby quelling proliferation and fibrosis, besides switching on the protective action of PPAR-*γ* signaling system [153].

#### 3.9.2. *In Vivo* Studies—Actually Nephroprotective Mechanisms

PPARs, important regulators of lipid and glucose metabolism, are ligand-activated nuclear transcription factors demonstrated to have a *protective* role in DN. *PPAR-γ* ameliorates hyperglycemia, insulin resistance, hypertension, and albuminuria, and inactivates diacylglycerol (DAG)/protein kinase C (PKC)/extracellular signal-regulated kinase (ERK) pathway with subsequent decrease in TGF-*β*1, fibronectin, and type IV collagen. It also dampens inflammation and oxidative stress and blocks atheroma-promoting mechanisms. *PPAR-α* activation reduces TGF-*β*1 and type IV collagen expression and prevents fatty acid build-up and lipid-induced toxicity in the diabetic kidneys [147].

Several herbal products are able to block the proliferation/fibrosis/sclerosis-inducing p38MAPK pathway, coupled with the activation of signaling pathways that may have protective effects, such as *PPAR-γ* (Danhong, an extract of *Salvia miltiorrhiza* and *Carthamus tinctorius* [154]) or *PI3K/Akt*. The upregulation of the latter by (-)-epigallocatechin-3-gallate was accompanied by a favorable effect on the RAS and on the oxidative status [155]. However, *Abelmoschus manihot* extract decreased the level of p-Akt, while still exerting a renoprotective effect associated with quenching of the p38MAPK pathway and anti-inflammatory, antifibrosing, and antioxidant effects [156]. An experiment done on *db/db* mice seems to indicate that Huangqi decoction shields the kidney from the consequences of diabetes through the agency of PI3K-Akt signaling. However, the activation of PI3K was associated with lower levels of the active form of Akt (p-Akt), which may explain why the positive effects of PI3K upregulation (higher expression of GLUT4 receptors that mediate intracellular glucose uptake) were not accompanied by the undesirable ones (protein synthesis and cellular proliferation driven by p-Akt) [36].

The flavonol myricetin activates *PPAR-α*, improves the biological and histopathological markers of kidney injury and fibrosis, reduces the levels of SREBPs, and favorably interferes with lipid metabolism [157]. As mentioned above, the antifibrosis effect of *Hypericum perforatum* was accompanied by the activation of *PPAR-γ* signaling pathway [31]. *Abelmoschus manihot* extract augmented the transcriptional activity of PPAR-*α* and PPAR-*γ*, improved the lipid status, and decreased the expression of inflammatory genes, ER stress, and c-Jun NH2-terminal kinase activation [104].

*EP4/Gs/AC/cAMP* signaling pathway (a Gs protein-coupled EP4 receptor that generates cAMP by activating AC) exerts a kidney-protective effect in diabetics ameliorating renal injury and slowing the progression of experimentally induced CKD [148]. At least some of the renoprotective effects of *berberine* are due to its ability to activate this pathway [158].

#### 3.9.3. Human Studies—Actually Nephroprotective Mechanisms

A systematic review and meta-analysis that took into account 66 studies involving 4785 participants concluded that *Astragalus membranaceus* (Huang Qi) increased the effectiveness of conventional therapies in lowering albuminuria, proteinuria, and serum creatinine levels, with no increase in adverse effects. However, methodological flaws resulted in a low quality of the available studies [159].

## 4. Strength of the Evidence in Various Areas

The evidence provided by *in vitro* and *in vivo* animal studies is abundant and strong. Much fewer are the human studies. We have found 12 references regarding clinical trials on herbal products in DN. Only 5 of them included information about putative action mechanisms. Two of them are high quality (J5), one on silymarin (decrease in albuminuria) [65], the other on Tangshen Formula (decrease in proteinuria, increase in eGFR) [124, 125]. The other three are low quality (J1): one on berberine (decrease in albuminuria) [95], one on *Dioscorea bulbifera* (decrease in proteinuria) [96], and one on *Salacia chinensis* (increased creatinine clearance, slowed CKD progression) [102]. Five other references describe study protocols on the efficiency of various traditional Chinese herbal medicines in DN [160–164]. The remaining two articles present the results of clinical trials that did not have as an objective the identification of action mechanisms. One is a moderate power randomized placebo-controlled trial demonstrating the ability of a Chinese herbal formula to decrease microalbuminuria [165]; the trial is of low-to-moderate quality, i.e., J2, as it was not double-blinded (only the participants were blinded) and no information on dropouts/withdrawals was provided. The other one was a moderate-powered double-blinded, randomized, high-quality (J5) clinical trial on saffron (*Crocus sativus* L.) but failed to show any significant change in kidney function parameters or albuminuria [166].

A recent meta-analysis of randomized placebo-controlled trials of Chinese herbal medicine for DN selected 20 studies including 2719 patients and concluded that plant products may favorably influence renal function and albuminuria beyond the beneficial effect of conventional therapy (mainly RAS inhibitors). However, the evidence was deemed of moderate-to-low quality. The medicinal plants most frequently employed in the various combinations were *Astragali radix*, *Rehmanniae Radix*, and *Rhei Radix et Rhizoma* [167]. The most studied herbal in DN seems to be *Astragalus membranaceus*, but the available studies are of low quality [159].

We may conclude that most of the existing studies are of inferior quality, but there are a few moderate- or high-quality ones proving that herbals may help prevent kidney injury and maintain kidney function in diabetic patients. Some of the most promising phytoceuticals are silymarin, *Astragalus membranaceus*, and Tangshen Formula. Given the vast experience with silymarin in the treatment of other (mainly liver) disorders, the wide availability of financially affordable silymarin containing preparations, and the lack of side effects, at least silymarin should be probably included in the standard treatment of diabetic patients, pending the completion of ongoing clinical trials on various other herbal combinations.

## 5. Concluding Remarks

**Figure 2:**
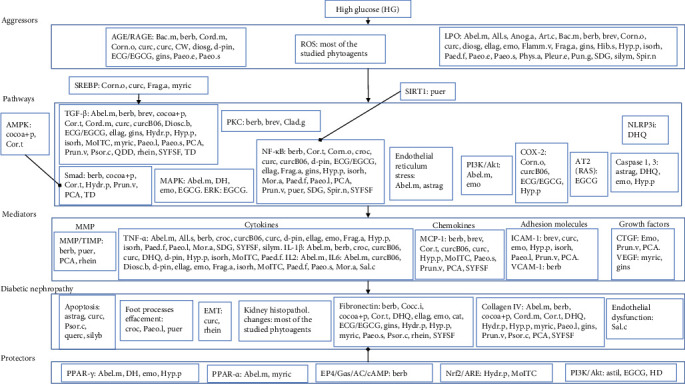
Graphical abstract: phytoagents active in diabetic nephropathy grouped according to their mechanism of action. Phytoagents: Abel.m: *Abelmoschus manihot*; All.s: *Allium sativum*; Anog.a: *Anogeissus acuminata*; Art.c: *Artemisia campestris*; astil: astilbin; astrag: astragaloside IV; Bac.m: *Bacopa monnieri*; berb: berberine; brev: breviscapine; cat: (+)-catechin; Chlor.p: *Chlorella pyrenoidosa*; Clad.g: *Cladophora glomerata*; Cocc.i: *Coccinia indica*; cocoa+p: cocoa enriched with polyphenols; Cor.t: *Coreopsis tinctoria*; Cord.m: *Cordyceps militaris*; Corn.o: *Cornus officinalis*; croc: crocin; curc: curcumin; curcB06: curcumin analogue B06; CW: coconut water; DH: Danhong; DHQ: dihydroquercetin; Diosc.b: *Dioscorea bulbifera*; diosg: diosgenin; d-pin: d-pinitol; ECG: (-)-epicatechin 3-O-gallate (*Camellia sinensis*); EGCG: (-)-epigallocatechin 3-O-gallate; ellag: ellagic acid; emo: emodin; Flamm.v: *Flammulina velutipes*; Frag.a: *Fragaria* × *ananassa*; gins: ginseng; HD: Huangqi decoction; Hib.s: *Hibiscus sabdariffa*; Hydr.p: *Hydrangea paniculata*; Hyp.p: *Hypericum perforatum*; isorh: isorhamnetin; MoITC: moringa isothiocyanate; Mor.a: *Morus alba*; myric: myricetin; Paed.f: *Paederia foetida*; Paeo.e: *Paeonia emodi*; Paeo.l: *Paeonia lactiflora*; Paeo.s: *Paeonia suffruticosa*; PCA: purple corn anthocyanins; Phys.a: *Physalis angulata*; Pleur.e: *Pleurotus eryngii*; Prun.v: *Prunella vulgaris*; Psor.c: *Psoralea corylifolia*; puer: puerarin; Pun.g: *Punica granatum*; QDD: Qidan Dihuang decoction; querc: quercetin; Rhe.r: *Rheum ribes*; Sal.c: *Salacia chinensis*; SDG: secoisolariciresinol diglucoside; silyb: silybin; silym: silymarin; Spir.n: *Spirogyra neglecta*; SYFSF: Shen-Yan-Fang-Shuai Formula; TD: Tangke decoction.

Many agents of plant origin have shown the ability to prevent DN, the mechanisms involved being, to a great extent, similar to those of the conventional drugs used for this aim: blocking RAAS, antioxidant, anti-inflammatory, and antiproliferative, preventing sclerosis/fibrosis, improving lipid profile, and activating protective pathways. Consequently, herbal medicines may emerge as healthy alternatives to the agents currently employed for slowing the progression of DN. The most important setback is the scarcity of human experiments, which is surprising and regrettable as these agents are generally devoid of side effects. The dearth of human studies is compounded by the low quality of most of the existing ones. Both these issues should be urgently addressed by future research in this area. A graphical abstract of this paper is provided as Figure 2.

## Figures and Tables

**Table 1:** Phytoceuticals effective in preventing diabetic nephropathy in animal models of DN. If not otherwise specified, the indicated dose was the daily dose and the route of administration was oral (generally by gastric gavage). If the strain of animals is not specified, that means that it was not specified in the cited article. The phytoceuticals are divided in plant materials, plant combinations, and plants. The plant materials (i.e., phytocompounds) are presented alphabetically according to the name of the medicinal plants of origin (which is put in parentheses, preceding the name of the plant material). For several phytocompounds there is no predominant plant source—those phytocompounds have, each, many sources. The studies that demonstrated actual nephroprotection are marked by “(NP)” in the “Type of study, model” column. The type of the study is also marked by the letters H=human study, T=*in vitro* study, V=*in vivo* study, T, V=*in vivo* and *in vitro* study also placed in parentheses in the “Type of study, model” column.

Herb/phytochemical, dose, and route of administration	Type of study, model	Findings	Ref.
Plant materials			
(*Arctium lappa*—fruit) arctigenin (a lignan), 40 mg/kg/d for 8 (eNOS−/− mice)/6 (*db/db* mice) weeks	(NP) (V) eNOS−/− mice, STZ; *db/db* (a strain of T2DM) mice	↓ albuminuria, KiHPCh; on podocytes: ↑ adhesion, ↓ motility, ↑ stability of actin cytoskeleton through Drebrin-1 (DBN1) dephosphorylation; ↑ protein phosphatase 2 A → ↓ p65 NF-*κ*B	[86]
(*Astragalus membranaceus*) astragaloside IV (a pentacyclic triterpenoid), (*in vivo*) 40 mg/kg/d for 12 weeks	(NP) (T, V) HG-cultured immortalized mouse mesangial cells SV40 MES 13; male KK-Ay mice with HFD-induced DM and male C57BL/6J mice	↑SIRT1 → ↓ p65 acetylation → ↓ NF-*κ*B → ↑ autophagy (↑ Beclin 1 and LC3 II) →↓ MC proliferation and activation; ↓ albuminuria, KiHPCh; ↓ *α*-SMA, FN, and collagen 4	[131]
(*Astragalus membranaceus*) astragaloside IV, 10 mg/kg for 8 weeks	(NP) (T, V) male SD rats, STZ; ER stress was induced in cultured human podocytes with tunicamycin	↓ albuminuria, s-creat, BUN, ECM expansion, phosphorylation of eukaryotic initiation factor 2*α*, protein kinase R-like ER kinase and JNK, ↓ glucose-regulated protein 78 and 150 kDa oxygen-regulated protein, ↓ apoptosis of podocytes, C/EBP homologous protein, cleaved caspase-3	[15]
(*Astragalus membranaceus*) astragaloside IV, 3, 6, 12 mg/kg/d for 8 weeks, (*in vitro*) 25, 50, and 100 *μ*M	(NP) (T, V) C57BL/6J mice, STZ; HG-cultured immortalized mouse podocytes.	↓ albuminuria, BUN, s-creat; ↓ KiHPCh; ↓ RAS (↓ renin); ↓ MCP-1, TNF-*α*; ↓ apoptosis; ↑ podocin and nephrin; ↓ ER stress (↓ GRP78, cleaved ATF6, p-PERK, p-IRE1, and CHOP); ↓ ER stress-induced apoptosis (↓ ATF6 and PERK, p-eIF2*α*, CHOP, p-IRE1*α*, p-JNK, ↓spliced X-box binding protein 1; ↓ cleaved caspase-12 and caspase-3); ↓ p-mTOR and p70S6 kinase; ↑ p-AMPK*α* (↑ AMPK*α* activation); ↑ autophagy; ↑ SERCA2	[103]
(*Berberis vulgaris*) berberine (a benzylisoquinoline alkaloid), 0.1 g tid for 24 months with a 2-week no-treatment interval every 5 months	(NP) (H) hypertensive T2DM patients with blood pressure and fasting plasma glucose adequately controlled by treatment. Low-quality (J1, i.e., the randomization method not described, the trial was not double-blinded, nor placebo-controlled, no description of withdrawals and dropouts) trial	↓ albuminuria, u-osteopontin, u-KIM-1, vascular cell adhesion molecule-1, C-reactive protein, MDA, u-8-hydroxy-2′-deoxyguanosine; ↑ SOD, total-antioxidant capacity, s-high molecular weight-adiponectin; improved renal hemodynamics	[95]
(*Berberis vulgaris*) berberine, 25 mg/kg for 20 weeks	(NP) (V) male Wistar rats, high-fat diet + STZ	↓ s-glu, lipids, albuminuria, NF-*κ*B, IL-1*β*, TNF-*α*, MCP-1, TGF-*β*-Smad3 signaling, fibronectin, collagen I, collagen 4, KiHPCh	[85]
(*Berberis vulgaris*) berberine, 50, 100, and 200 mg/kg for 8 weeks	(NP) (V) male SD rats, STZ	↓ s-glu, KW/BW, proteinuria, BUN, s-creat, KiHPCh, AGEs, RAGE, p-PKC-*β*, TGF-*β*1; ↑ BW	[118]
(*Berberis vulgaris*) berberine, 50, 100, and 200 mg/kg for 8 weeks	(NP) (V) male SD rats, STZ	↓ s-glu, proteinuria, BUN, s-creat, KW, TGF-*β*1, fibronectin, collagen 4, ECM accumulation; ↑ ratio of MMP-2/TIMP-2 and MMP-9/TIMP-1, ECM degradation	[117]
(*Berberis vulgaris*) berberine, 50, 100, and 200 mg/kg for 8 weeks	(NP) (V) male SD rats, high-sugar, and high-fat diet + STZ	↓ proteinuria, BUN, s-creat, KiHPCh; ↑ EP4 and G*α*s, cAMP	[158]
(*Berberis vulgaris*) berberine, 50, 100, and 200 mg/kg/d 8 weeks	(NP) (V) male SD rats, STZ + high-fat diet	↓s-glu, proteinuria, albuminuria, BUN, s-creat, TG, TC, LDL-C, collagen 4, TGF-*β*1, GRK2, GRK3 ↑ HDL-C, cAMP, GRK6	[121]
(*Berberis vulgaris*) berberine, *in vivo*: 100 mg/kg for 8 weeks, *in vitro*: 5, 10, 30, 60, 90, 120, and 240 *μ*M	(NP) (T, V) male SD rats, high-glucose, and high-fat diet + STZ; HG-incubated renal cortical cells	↓ KW/BW, proteinuria, BUN, s-creat, PGE2, renal prostaglandin E2 receptor 1, KiHPCh, G*α*q, proliferation of GMCs, cytoplasmic calcium in glomerular mesangial cells	[122]
(*Berberis vulgaris*) berberine: 10, 30, 60, 90 *μ*M	(T) normal rat renal tubular epithelial (NRK-52E) and human kidney proximal tubular (HK-2) cells	↓ apoptosis (↓ cytochrome c, Bax, caspase-3 and caspase-9); ↑ PI3K/Akt; ↑ Nrf2, HO-1; ↓ mitochondrial function (↓ mitochondrial membrane potential); ↓ ROS production; ↑ GSH, SOD	[44]
(*Berberis vulgaris*) berberine: 2.5 or 5 *μ*M	(T) HG-cultured immortalized mouse (MPC5) podocytes	↓ apoptosis; ↑ nephrin and podocin; ↑ AMPK activation; ↓ mTOR →↑ autophagy	[136]
(*Berberis vulgaris*, *Coptis chinensis*) berberine: 30 *μ*M	(T) HG-cultured NRK-52E and HK-2 cells exposed to hypoxia	↑ hypoxia/HG-induced HIF-1*α* expression and nuclear translocation; ↑ p-Akt (PI3K/Akt) and HIF-1*α* →↓apoptosis (↑ Bcl-xL, ↓ Bax, cytochrome C, cleaved caspase-3, cleaved caspase-9)	[45]
(*Camellia sinensis*) (-)-epigallocatechin-3-gallate (a type o catechin), 50 and 100 mg/kg for 8 weeks	(NP) (V) *db/db* mice	↓ s-glu, area under the curve at OGTT, proteinuria, u-8-hydroxy-2′-deoxyguanosine, angiotensin II, angiotensin II type 1 receptor, p22-phox, p47-phox, p-extracellular regulated protein kinases (p-ERK1/2), p-p38MAPK, KiHPCh; ↑ insulin, p-PI3K, p-Akt	[155]
(*Camellia sinensis*) green tea (+)-catechin, 35 mg/d for 12 weeks	(NP) (V) male SD rats, STZ	↓ albuminuria and s-creat; ↑ u-creat and CrCl; ↓ endothelin-1, LPO, ALT, and expression of fibronectin; ↑ free thiols	[46]
(*Camellia sinensis*) green tea catechins, 5 mg, administered in the drinking water for 12 weeks	(NP) (V) male SD rats, STZ	↓ albuminuria, interstitial fibrosis	[3]
(*Camellia sinensis*) green tea leaves polyphenols: (-)-epicatechin 3-O-gallate (ECG) and (-)-epigallocatechin 3-O-gallate (EGCG), ECG: 10 and 20 *μ*M/kg/d; EGCG: 25, 50, and 100 mg/kg for 50 days	(NP) (V) male Wistar rats, ischemia-reperfusion + lipopolysaccharide	ECG: ↓ 3-NT, ONOO−, ∙OH, MPO, protein nitration, LPO, s-uric, BUN, s-creat, proteinuria; ↑ SOD, CAT, GPx, GSH; EGCG: ↓ s-glu, proteinuria, albuminuria, TC, TG, BUN, s-creat, glomerular and tubulointerstitial injury, AGEs, LPO, iNOS, COX-2, NF-*κ*B, p-I*κ*B-*α*, TGF-*β*1, fibronectin, KiHPCh; ↑ s-protein, s-albumin, CrCl	[81]
(*Cocos nucifera*) coconut water, 3 mL/kg; caffeic acid (an ingredient of coconut water), 10 and 15 mg/kg, pretreatment for 5 days and posttreatment for 6 days	(NP) (V) male Wistar rats, alloxan	↓ s-glu, HbA1c, s-urea, peroxidase activity, Amadori product, nonprotein thiols	[61]
(*Crocus sativus*) crocin (a carotenoid), 0.1, 0.5, and 1 *μ*M pretreatment	(T) HG- (15 or 25 mM-) cultured immortalized mouse podocytes	↓ROS production, IL-1*β*, IL-8, IL-10, TNF-*α*, p-I*κ*B*α*; ↑nephrin, podocin, CD2ap (markers of slit diaphragms reduced by HG), SOD	[73]
(*Curcuma longa—*roots) curcumin (a diarylheptanoid polyphenol), 10 *μ*M	(T) AGE-treated rat kidney tubular epithelial cell line NRK-52E	↑ PI3k/AKT → ↑ autophagy (↑ autophagic vacuolization (LC3, ratio LC3II/LC3I and Beclin)) → ↓ AGEs-induced apoptosis (↓ Bax and apoptosis-inducing factor, cleaved caspase-3 and caspase cascade activation)	[132]
(*Curcuma longa*) curcumin, 100 mg/kg for 12 weeks	(NP) (T, V) male Wistar rats, STZ; HG-cultured podocytes	↓ KW/BW, proteinuria, EMT of podocytes, ECM expansion, GS, GBM thickening, podocyte foot processes effacement, renal fibrosis, caveolin-1 Tyr(14) phosphorylation; ↑ CrCl, stabilization of caveolin-1 and *β*-catenin	[130]
(*Curcuma longa*) curcumin, 1.5 mg/kg for 8 weeks	(NP) (V) male Wistar rats, STZ + nicotine	↓ TC, TG, PL, MDA, *γ*-glutamyltranspeptidase, s-uric, s-urea, s-creat, vimentin, desmin, SREBP-1, iNOS, TGF-*β*1, KiHPCh, pancreatic histopathological changes; ↑ HDL, SOD, GPx, synaptopodin, connexin 43, erythropoietin	[108]
(*Curcuma longa*) curcumin, 100 mg/kg for 8 weeks	(NP) (V) male SD rats, STZ	↓ s-glu, BUN, proteinuria, macrophage infiltration, TNF-*α*, IL-1*β*, degradation of I*κ*B*α*, NF-*κ*B, ICAM-1, MCP-1, TGF-*β*1 expression; ↑ CrCl, BW	[79]
(*Curcuma longa*) curcuminoids (curcumin and demethoxycurcumin), 0.01-1 nM	(T) rat mesangial cell line HBZY-1	↓ ROS generation, MDA, apoptosis (increased by AGEs); ↑ SOD (decreased by AGEs)	[41]
(*Cyclocarya paliurus*) triterpenic acids-enriched fraction	(NP) (T, V) male SD rats, STZ; HG-induced HK-2 cells	↓ albuminuria, s-creat, BUN, KiHPCh; ↑ p-AMPK → ↓ p-mTOR; AMPK activation → ↑ autophagy → ↓ apoptosis (↓ caspase-3)	[142]
(*Dioscorea* spp.) diosgenin (a phytosteroid sapogenin), 5, 10, and 20 mg/kg for 28 days	(NP) (V) male Wistar rats, STZ	↓ s-glu, polyphagia, polydipsia, TC, TG, s-creat, proteinuria, KW/BW, MDA, MPO, AGEs, KiHPCh including GBM thickening; ↑ BW, CrCl, HDL-C, SOD, CAT, GSH	[62]
(*Elaeis guineensis/Oryza sativa*) palm oil and rice bran oil—tocotrienol rich fraction, 200 mg/kg, for 8 weeks	(NP) (V) male Wistar rats, STZ	↓ s-glu, HbA1c, s-creat, BUN, proteinuria, NO, TBARS, MDA; ↑ CrCl, SOD, CAT	[50]
(*Erigeron breviscapus*) breviscapine (a trihydroxyflavone, a.k.a. scutellarin), 20 mg/kg for 8 weeks	(NP) (V) male Munich-Wistar rats, STZ	↓ albuminuria, MDA, PKC, TGF-*β*1, ICAM-1, MCP-1, macrophage infiltration, glomerular hypertrophy, tubulointerstitial injury; ↑ SOD, CAT, GPx	[51]
(*Erigeron breviscapus*) breviscapine, 20 mg/kg for 8 weeks	(NP) (V) male Munich-Wistar rats; STZ	↓ albuminuria, KiHPCh, 3-NT, MDA, PKC, TGF-*β*1	[52]
(*Glycine max*) d-pinitol (a cyclitol), 50 mg/kg for 30 days	(NP) (V) male Wistar rats, STZ	↓ s-urea, s-uric, s-creat, AGEs, TNF-*α*, IL-1*β*, IL-6, NF-*κ*Bp65, nitrite, LPO, hydroperoxides, protein carbonyls, KiHPCh; ↑ s-protein, SOD, CAT, GPx, GST, GRed, vitamin E, vitamin C, GSH	[2]
(*Hypericum perforatum*) astilbin, 10 and 20 *μ*M	(T) HK-2 cells	↓ autophagy, apoptosis, caspase-3, Bax; ↑ Bcl-2, p-Akt	[140]
(*Linum usitatissimum*) secoisolariciresinol diglucoside (the major lignan in flaxseed), 10 and 20 mg/kg for 4 weeks	(NP) (V) male SD rats, STZ + high-fat diet	↓ s-glu, fructosamine, s-creat, BUN, MDA, NO, NF-*κ*B, TNF-*α*, iNOS; ↑ insulin, GSH, SOD, antiapoptotic markers (survivin, Bcl-2)	[88]
Curcumin analogue (B06), 0.2 mg/kg for 6 weeks	(V) male SD rats and Institute of Cancer Research (ICR) mice, STZ	↓ TNF-*α*, COX-2, TGF-*β*, MCP-1, IL-6, IL-12, IL-1*β*, iNOS, JNK/NF-*κ*B signaling, nitrite, macrophage infiltration, KiHPCh	[66]
(many sources) dihydroquercetin (a flavanonol a.k.a taxifolin), *in vivo*: 100 mg/kg for 12 weeks; *in vitro*: 5, 10, 20, 40, and 80 *μ*M for 72 h	(NP) (T, V) SD rats, high-fat diet + STZ; HG-cultured rat kidney mesangial cells (HBZY-1) and human proximal renal tubular epithelial cells (HK-2 = human kidney 2)	*In vivo*: ↓ albuminuria, s-creat, s-glu, LDL-C, TC, KW/BW, KiHPCh including cell proliferation, ROS generation; *in vitro*: ↓ activation of NLRP3 inflammasome, ↓ cleaved caspase-1, IL-1*β*, fibronectin, collagen 4	[48]
(Many sources) ellagic acid (a phenolic acid), *in vivo*: 20 and 40 mg/kg, for 16 weeks; *in vitro*: 5 *μ*M	(NP) (T, V) male Wistar albino rats, high-fat diet + STZ; HG-cultured rat NRK 52E proximal tubular epithelial cells	↓ s-glu, polyphagia, BW, HbA1c, insulin resistance, TC, LDL-C, VLDL-C, FFA, TG, GOT, GPT, ALP, MDA, s-creat, BUN, proteinuria, KiHPCh, NF-*κ*Bp65, TGF-*β*1, fibronectin, IL-1 *β*, IL-6, TNF-*α*; ↑ HDL-C, GSH, GPx, GR, SOD, CAT, CrCl	[37]
(Many sources) ferulic acid (a hydroxycinnamic acid), (*in vitro*) 0–200 *μ*M, (*in vivo*) 10, 30, 50, and 70 mg/kg/d for 8 weeks	(NP) (T, V) male Wistar rats, STZ; HG-induced normal rat kidney epithelial-like (NRK-52E) cells	↓ gluc, BUN, s-creat, s-uric, albuminuria; ↓ KiHPCh; ↓ ROS, NO, protein carbonyl, MDA, ↑ SOD2, catalase, ↑ GSH/GSSG ratio; ↓ AGEs, ↓ xanthine oxidase, ↓ hydroxyproline (fibrosis); ↓ MAPK (↓ phosphorylation of p38, JNK, and ERK1/2 MAPKs); ↓ neutrophil infiltration (↓ MPO); ↓ TNF-*α*, IL-1*β*, IL-6, ↓ MCP-1, ICAM-1, VCAM-1; ↓ NF-*κ*B (↓ I*κ*B*α* degradation), iNOS, and COX-2; ↑ autophagy (↑ beclin-1 and LC3-II, ↓ p62); ↓ mitochondrial dysfunction (↑ mitochondrial dehydrogenases); ↓ apoptosis (↓ cytosolic cytochrome c, Bax/Bcl-2 ratio and cleaved caspase-9, ↓ extrinsic pathway of apoptosis: ↓ Fas-L, Fas-R, caspase-3 activation, PARP cleavage)	[145]
(Many sources) isorhamnetin (a methoxylated flavonol), *in vivo*: 50 and 150 mg/kg for 12 weeks; *in vitro*: 5 and 10 *μ*M	(NP) (T, V) male SD rats, high-fat diet + STZ; glomerular mesangial cells, lipopolysaccharide	↓ u-osteopontin, u-KIM-1, albuminuria, NF-*κ*Bp65, p-NF-*κ*Bp65, p-I*κ*B*α*, NF-*κ*Bp65 DNA-binding activity; TNF-*α*, IL-1*β*, IL-6, ICAM-1, TGF-*β*1, MDA; ↑ SOD	[87]
(Many sources) mangiferin (a xanthone), (*in vivo*) 12.5, 25, or 50 mg/kg/d for 12 weeks; (*in vitro*) 50, 10, and 5 *μ*M	(NP) (T, V) male SD rats, STZ; HG-cultured immortalized mouse podocytes (MPC5)	↓ albuminuria, ↓ glomerular ECM expansion, ↑ nephrin; ↑ autophagy; ↑ p-AMPK; ↓ p-mTOR; ↑ p-ULK1	[139]
(Many sources) myricetin (a flavonol), 1.0 mg/kg for 12 weeks	(NP) (V) male albino Wistar rats, STZ + Cd	↓ albuminuria, TC, TG, FFA, PL, LDL, VLDL, 3-HO 3-methylglutaryl coenzyme A reductase, SREBP-1a, SREBP-1c, and SREBP-2, TGF-*β*1, VEGF, fibronectin, collagen 4, pancreatic histopathological changes, KiHPCh; ↑ PPAR-*α*, HDL, lipoprotein lipase, lecithin cholesterol acyl transferase	[157]
(Many sources) quercetin (a flavonol), 10 mg/kg for 4 weeks	(NP) (V) adult C57BL/6J mice, STZ	↓ polyuria, s-glu, TG, proteinuria, s-uric, s-urea, s-creat, superoxide anions, KiHPCh, apoptosis	[49]
(many sources) resveratrol (a polyphenolic phytoalexin and a stilbenoid) (*in vivo*) 100 mg/kg/d intragastric for 12 weeks, (*in vitro*) 1 *μ*M, 10 *μ*M, 100 *μ*M	(NP) (T, V) male *db/db* mice; HG-induced conditionally immortalized mouse podocytes	↓ albuminuria, s-creat, KiHPCh; ↑ LC3-II/LC3-I and synaptopodin, ↓ cleaved caspase-3; ↑ miR-18a-5p (via targeting atactic telangiectasis mutation) → ↓ apoptosis (↓ cleaved caspase-3) and ↑ autophagy (↑ LC3-II/LC3-I)	[133]
(Many sources) resveratrol (*in vivo*) 10 mg/kg/d by oral gavage for 12 weeks, (*in vitro*) 5, 10, 15 *μ*M	(NP) (T, V) *db/db* mice, HG-cultured human podocytes	↓ microalbuminuria, s-creat, BUN; ↓ KiHPCh, ↑ nephrin, ↓ apoptosis (↓ cleaved caspase-3 and Bax); ↑ autophagy (↑ LC3-II and synaptopodin, ↑ Atg5, ↓ p62, ↑ number of autophagosomes); ↓ miR-383-5p → ↑ autophagy → ↓ apoptosis of podocytes	[168]
(Many sources) resveratrol (*in vivo*) 5 mg/kg/d mixed with the food for 4 months, (*in vitro*) 5, 10, 15 *μ*M	(NP) (T, V) male SD rats, HG-cultured NRK-52E (rat renal proximal tubular cells)	↓ TNF-*α*, IL-6, IL-1*β*, IL-10; ↓ s-cystatin C, albuminuria, HbA1c, s-creat, BUN; ↓ 8-OHdG; ↑ SIRT1; ↑ NAD; ↑ autophagy (↑ related genes: Atg5, Atg7, Foxo3, SIRT1, Bnip3); ↑ LC3II, ↑ ratio LC3II/LC3I; ↑ hypoxia-induced autophagy; ↑ Hif1a	[144]
(*Moringa oleifera*) moringa isothiocyanate, 1.25, 2.5, and 5 *μ*M	(T) HG-cultured human renal proximal tubule HK-2 cells	↑ Nrf2-antioxidant responsive elements and the downstream genes NAD(P)H:quinone oxidoreductase 1, HO-1, and glutamate cysteine ligase catalytic subunit; ↓ iNOS, IL-6, IL-1*β*, MCP-1, IL-1A, ROS production, TGF-*β*1	[42]
(*Paeonia suffruticosa*) paeoniflorin (a terpene glycoside) and oxypaeoniflora (a lactol), 0.01-100 *μ*M	(T) coculture system of mesangial cells HBZY-1 and macrophages	↓ macrophage migration, IL-6, MCP-1 (increased by AGEs); ↑ GPx and CAT (decreased by AGEs)	[40]
(*Panax ginseng*) 20(S)-ginsenoside Rg(3), 5, 10, 20 mg/kg for 15 days	(V) rats, STZ	↓ polydipsia, polyuria, s-glu, glycosylated protein, TBARS, ameliorates renal dysfunction	[169]
(*Plantago asiatica*) hispidulin (a monomethoxyflavone), 2.0, 5.0 *μ*M	(T) HG-cultured immortalized mouse podocytes (MPC5)	↓ EMT (↑ nephrin and podocin), ↓ apoptosis (↓ caspase-3), ↑ autophagy by regulating Pim1/p21/mTOR (↓ p-mTOR mainly dependent on Pim1)	[137]
(*Pueraria lobata*) puerarin (an isoflavone), 100 mg/kg for 7 days, i.p.	(NP) (V) male Wistar rats, STZ	↓ KiHPCh including podocyte foot processes effacement, proteinuria, ROS production, S-nitrosylation of proteins, MMP-9; ↑ podocyte slit diaphragm proteins (nephrin, podocin)	[60]
(*Pueraria lobata*) puerarin, *in vivo*: 20 mg/kg for 8 weeks; *in vitro*: 5%	(NP) (T, V) eNOS−/−) mice + STZ (an accelerated DN model); HG-cultured murine podocytes	↓ albuminuria, KiHPCh, oxidative stress, superoxide, NOX4; ↑ SIRT1, SIRT1-mediated deacetylation of NF-*κ*B	[76]
(*Rheum officinale*) rhein (a dihydroxyanthraquinone), 25, 50, and 100 *μ*g/mL	(T) HG-cultured immortalized proximal tubular cells HK-2	↓ integrin-linked kinase, EMT, MMP-9/TIMP-1 ratio	[127]
(*Rheum officinale*, rhubarb) rhein, 150 mg/kg for 12 weeks	(NP) (V) *db/db* (a strain of T2DM) mice	↓ albuminuria, ECM, TGF-*β*1, fibronectin, TC, TG, LDL-C, ApoE	[113]
(*Rheum palmatum—*root) emodin (a trihydroxyanthraquinone), the main active component of rhubarb, 100 mg/kg once every 3 days for 3 weeks	(NP) (V) female adult Wistar rats, STZ	↓ s-glu, KW, albuminuria, s-creat, tubulointerstitial injury, IL-6, TNF-*α*, MDA, ICAM-1, Bax, caspase-3; ↑ SOD, p-Akt, and p-glycogen synthase kinase 3*β*	[94]
(*Rheum palmatum*) emodin, 30 and 60 *μ*M	(T) HG-cultured rat mesangial cell line (HBZY-1)	↓ cell proliferation and stops cell cycle progression; ↓ fibronectin, p-p38MAPK, p-cAMP response element-binding protein, CTGF; ↑ PPAR-*γ*	[153]
(*Silybum marianum*) silybin (a flavonolignans), *in vivo*: 100 mg/kg i.p. for 6 weeks; *in vitro*: 10 *μ*M	(NP) (T, V) OVE26 mice (a model of T1DM and DN); HG- (25 mM) cultured mouse podocytes	↓ NOX4, superoxide production, podocyte apoptosis, albuminuria	[43]
(*Silybum marianum*) silymarin, three 140 mg tablets for 3 months	(NP) (H) randomized, double-blind, placebo-controlled trial on patients with T2DM, eGFR >30 mL/min/1.73 m^2^ and albuminuria >300 mg/24 h (despite maximal renin-angiotensin system inhibitor therapy for ≥6 months). High-quality (J5) trial	↓ albuminuria, TNF-*α*, MDA	[65]
(*Theobroma cacao*) cocoa enriched with polyphenols, 24 mg/kg for 16 weeks	(V) spontaneously hypertensive rats, STZ	↓ TGF-*β*1, p-Smad2, collagen 4, fibronectin, NOX4; ↑ p-AMPK or activation of AMPK; effects abolished by AMPK blockade	[110]
(*Tripterygium wilfordii—*root bark) tripterygium glycosides	(T) HG-induced *db/db* mouse podocytes	↓ apoptosis, ↑ nephrin and podocin, ↓ *β*-arrestin-1, ↑ autophagy (↑ LC3-II and LC3-II/LC3-I ratio, ↓ p62)	[138]
(*Tripterygium wilfordii—*roots) celastrol (a triterpenoid), 0.1, 0.2, 0.6, 1.0, 1.5, and 2 *μ*M	(T) HG-cultured mouse podocytes	↓ apoptosis, LDH, ROS; ↓ IL-1*β*, TNF-*α*, IL-6; ↓ insulin resistance; ↑ nephrin; ↑ autophagy (↑ LC3 II and Beclin-1, ↓ p62); ↑ HO-1	[170]
(*Tripterygium wilfordii*) triptolide (heteroheptacyclic epoxide, gamma-lactam, and diterpenoid), 200 *μ*g/kg/d for 12 weeks	(NP) (T, V) male SD rats, high-fat diet + STZ; HG-cultured human mesangial cells	↑ autophagy, ↓ fibrosis (fibronectin, collagen 4) by means of miR-141-3p/PTEN/Akt/mTOR (↑ PTEN, ↓ p-Akt, ↓ p-mTOR)	[141]
(*Zea mays*) purple corn anthocyanins (mainly cyanidin 3-glucoside and cyanidin-3-(6″-malonylglucoside)), 1-20 *μ*g/mL	(T) HG-cultured human renal mesangial cell	↓ CTGF, ICAM-1, MCP-1, TGF-*β*, collagen 4, TIMP-2, TGF-*β*-Smad signaling, ↓ NF-*κ*B translocation, mesangial hyperplasia and inflammation; ↑membrane MMP-1, ECM degradation	[70]

Plant combinations			
Huangqi decoction (an extract from 7 herbs: astragalus, poria, trichosanthes roots, ophiopogon, schisandra, licorice and rehmannia), 1.08, 0.36, and 0.12 g/kg for 14 weeks	(NP) (V) male *db/db* mice	↓ s-glu, s-glu increase at OGTT, polyphagia, polydipsia, polyuria, BW, insulin resistance, HbA1c, albuminuria, s-creat, BUN, KiHPCh, p-Akt, GLUT (glucose transporter)1; ↑ GFR, p-insulin receptor (IR), p-IR substrate, p-PI3K, GLUT4	[36]
Shen-Yan-Fang-Shuai Formula (SYFSF)—a traditional Chinese formula composed of *Astragali radix*, *Radix angelicae sinensis*, *Rheum officinale*, and four other herbs, 11.4 g/kg for 8 weeks	(NP) (T, V) Wistar rats uninephrectomy + high-fat diet + STZ; high-glucose cultured rat renal mesangial cell line (HBZY-1)	↓ albuminuria, TC, TG, s-creat, interstitial expansion, GS, MCP-1, TGF-*β*1, collagen 4, fibronectin, TNF-*α*, p-NF-*κ*Bp65	[80]
Tangke decoction, 18 mg/kg for 12 weeks (prevention) or for 8 weeks (treatment)	(NP) (V) male SD rats, STZ	↓ KW, KW/BW, s-glu, proteinuria, albuminuria, TGF-*β*1, Smad4, KiHPCh; ↑BW	[116]
Tangshen Formula (*Astragali radix*, *Euonymi ramulus*, *Rehmanniae radix*, *Aurantii fructus*, *Corni fructus*, *Rhei radix et rhizoma*, *Notoginseng radix*), (*in vivo*) 2.4 g/kg/d for 12 weeks, (*in vitro*) 500, 750, and 1000 *μ*g/mL	(NP) (T, V) C57BLKS/J *db/db* mice, NRK52E cells	↓ proteinuria, KiHPCh; ↓ promyelocytic leukemia zinc finger protein, ↓ collagen 3 accumulation; ↑ autophagy; ↓ cell proliferation	[123]
Tangshen Formula 8 g × 2/d for 24 weeks	(NP) (H) six-center randomized, double-blind, placebo-controlled trial on 180 patients with DKD. High-quality (J5) trial	↓ proteinuria, ↑ eGFR, ↓ liver-type fatty acid binding protein	[124, 125]
Qidan Dihuang decoction, *Radix Astragali* 3.15 g/kg, *Radix Salviae Miltiorrhizae* 1.56 g/kg, *Radix Rehmanniae* 1.56 g/kg, Chinese yam 1.56 g/kg, liquorice 0.5 g/kg for 8 weeks	(NP) (V) male SD rats, STZ	↓ s-creat, proteinuria, KiHPCh, *α*-smooth muscle actin, TGF-*β*, renin, AT1	[119]

Plants			
*Abelmoschus manihot* extract = Huangkui capsule, 0.75 and 2 g/kg for 8 weeks	(NP) (V) male SD rats; unilateral nephrectomy + STZ	↓ KW, albuminuria, BUN, s-uric, renal fibrosis/GS, MDA, 8-hydroxy-2′-deoxyguanosine, NOX4, p-p38MAPK, p-Akt, TGF-*β*1, TNF-*α*; ↑ BW, SOD	[156]
*Abelmoschus manihot* extract = Huangkui capsule, 300, 175, and 75 mg/kg for 12 weeks	(NP) (V) male SD rats; unilateral nephrectomy + STZ	↓ TG, TC, TNF-*α*, IL-6, IL-1*β*, IL-2, endoplasmic reticulum stress, JNK, proteinuria, KiHPCh including ECM expansion and GS, TGF-*β*, collagen 4; ↑ s-albumin, PPAR*α*, PPAR*γ*	[104]
*Abelmoschus manihot* flower or leaf extracts, 100 mg/kg/day by oral gavage for 5 weeks	(NP) (V) mice after unilateral nephrectomy + high-fat diet + STZ	↓ gluc, s- creat, BUN, albuminuria; ↓ KiHPCh; ↑ autophagy- (SIRT-1, ATG5, ATG12), ↑ autophagy dynamics (↑ LC3B-II, ↓ p62); ↓ mitochondrial fragmentation; ↓ hepatic injury (ALT, AST, hepatic necrosis, liver lipid accumulation); ↓ TNF-*α*, IL-6; NF-*κ*B (p-I*κ*B*α*)	[143]
*Allium sativum*–aged garlic extract, 500 mg/kg for 12 weeks	(NP) (V) male albino Wistar rats, STZ	↓ BW, polyuria, HbA1c, albuminuria, s-creat, BUN, TG, TC, LDL-C, KiHPCh; ↑ HDL-C, u-creat, u-urea	[171]
*Allium sativum* (garlic) aqueous extract, 2 g/kg/d	(NP) (V) Wistar rats, STZ + nicotinamide	↓ TNF-*α*, s-glu, s-uric, s-urea, MDA, NO, total oxidant status	[28]
*Anogeissus acuminata*, 100 and 300 mg/kg for 8 weeks	(NP) (V) male Wistar rats, STZ	↓ s-glu, s-creat, BUN, MDA, proteinuria, KW/BW; ↑ urinary volume (reduced by diabetes) (*sic*); ↑ GSH, CAT	[56]
*Artemisia campestris*, 200 mg/kg for 3 weeks, i.p.	(NP) (V) male Wistar rats, alloxan	↓ s-glu, s-urea, s-creat, s-uric, MDA, NO, advanced oxidation protein products, KiHPCh ↓SOD, CAT, GPx (*sic*); ↑ insulin, CrCl, GSH	[55]
*Artemisia sieberi*—essential oil extract, 100 mg/kg for 90 days	(NP) (V) male albino Wistar rats, STZ + Cd	↓ s-glu, glucagon, TC, TG, LDL-C, ESR, s-urea, s-uric, s-creat; ↑ total protein, albumin, insulin, HDL-C, neutrophil count, and hematocrit	[29]
*Bacopa monnieri*—alcohol and hydroalcohol extract, 100, 200, and 400 mg/kg; stigmasterol from *B. monnieri* extract: 5 and 10 mg/kg for 45 days	(NP) (V) male Wistar rats, nicotinamide + STZ	↓ s-glu, s-uric, s-creat, lipid, AGEs, TBARS. ↑ SOD, GSH	[30]
*Boerhaavia diffusa*—ethanolic extract, 500 mg/kg for 30 days	(V) female albino Wistar rats, alloxan	Maintained the ionic balance and renal Na+-K+ ATPase activity, ↓ s-glu, LPO; ↑ GPx, CAT, SOD, GSH	[172]
*Chlorella pyrenoidosa*, 100 mg/kg for 90 days	(NP) (V) male albino Wistar rats, STZ + Cd	↓ s-glu, s-creat, BUN, TC, VLDL-C, LDL-C, TG, FFA, PL, KiHPCh; ↑ insulin, HDL-C	[173]
*Cladophora glomerata* extract, 1 g/kg 2-4 times a day for 12 weeks	(NP) (V) male Wistar rats, high-fat diet + STZ	↓ s-glu, TG, insulin resistance, PKC-*α*, KiHPCh; ↑ Oat1 and 3 functions, PKC-*ζ*	[34]
*Coccinia indica*, fruits and leaves, diet supplement 10% and 5% for 2 months	(NP) (V) male Wistar rats, STZ	↑ BW; ↓ s-glu, glucosuria, albuminuria; ↓ glomerular filtration rate; ↓ KW/BW; ↓ laminin, fibronectin; ↑ CAT, GPx, GRed, GST	[111]
*Cordyceps militaris*—combination of powders of fruiting bodies and mycelia, 360 mg/kg for 8 weeks	(NP) (V) C57BL/6J mice, high-fat diet + nicotinamide + STZ	↓ s-glu, s-creat, TG, TC carboxymethyl lysine (an AGE), TGF-*β*1, KW/BW, KiHPCh, collagen 4	[114]
*Coreopsis tinctoria* ethyl acetate extract, 25, 50, 100, and 150 mg/mL; marein (the main ingredient), 100, 200, 300, and 400 *μ*M	(T) rat mesangial cells (HBZY-1)	↓ mesangial cell proliferation and fibrogenesis, collagen 4, fibronectin, and TGF-*β*1, TGF-*β*-Smad signaling, p-Smad2/3 and Smad4, NF-*κ*B, NF-*κ*B P-65, MCP-1; ↑ p-AMPK	[72]
*Cornus officinalis* fruit; morroniside, loganin, and 7-O-galloyl-D-sedoheptulose, the main active compounds, morroniside 20 and 100 mg/kg for 8 weeks; loganin 20 or 100 mg/kg for 8 weeks; 7-O-galloyl-D-sedoheptulose 20 or 100 mg/kg for 8 weeks	(NP) (V) *db/db* (a strain of T2DM) mice	Morroniside: ↓ TG, ROS, TBARS, AGEs, SREBP-1 and SREBP-2, NF-*κ*B. Loganin: ↓ polyphagia, s-glu, TG, TBARS, N*ε*-(carboxymethyl)lysine (CML, an AGE) accumulation, ↑ GSH/GSSG ratio. 7-O-galloyl-D-sedoheptulose: ↓ glu, TG, s-creat, BUN, SREBP-1 (no effect on SREBP-2), ROS, TBARS, NF-*κ*B, COX-2, iNOS, AGEs (CML and N*ε*-(carboxyethyl)lysine (CEL))	[47]
*Cydonia oblonga* fruit aqueous extract, 80, 160, and 240 mg/kg for 6 weeks	(NP) (V) male SD rats, STZ	↓ TG, TC, LDL-C, ALT, AST, ALP, s-urea, s-creat; ↑ HDL-C	[174]
Danhong (extracted from *Salvia miltiorrhiza* and *Carthamus tinctorius*) injection, 2 mL/kg for 2 weeks, i.p.	(NP) (V) male SD rats; unilateral nephrectomy + high-fat diet + STZ	↓ BUN, s-creat, cystatin C, proteinuria, TC, LDL-C, p38MAPK, KiHPCh; ↑ HDL-C, PPAR*γ*, uncoupling protein-1 (a downstream signaling molecule)	[154]
*Dioscorea bulbifera*, 500 mg bid for 6 months	(NP) (H) hospital-based single-center prospective open-label randomized case-control interventional study on patients with DN with proteinuria >500 mg or albuminuria >300 mg/d, s-creat ≤2.5 mg/dL and hypertension controlled with a single drug. Low-quality (J1, i.e., the randomization method not described, the trial was not double-blinded, nor placebo-controlled, no description of withdrawals and dropouts) trial	↓ systolic and diastolic blood pressure, s-glu, LDL, proteinuria, TGF-*β*, IL-6, C-reactive protein	[96]
*Flammulina velutipes* polysaccharides, 800, 400, and 200 mg/kg for 15 days	(NP) (V) male Kunming mice, STZ	↓ s-glu, s-creat, BUN, s-albumin (*sic*), MDA, KW/BW, KiHPCh; ↑ BW, SOD, CAT, GPx	[53]
*Fragaria × ananassa*, (strawberry), aqueous, hydroalcoholic, and alcoholic extracts, 2 g/kg for 4 weeks	(NP) (V) albino Wistar rats, nicotinamide + STZ	↓ s-glu, AST, ALT, ALP, TC, LDL, VLDL, TG, s-creat, MDA, several fatty acid synthesis genes, SREBP, NF-*κ*B, IL6, TNF-*α*, KiHPCh; ↑ CAT, liver PPAR-*γ*, HDL	[89]
*Hibiscus sabdariffa*, 250 mg/kg for 7 weeks	(NP) (V) SD rats 5/6 nephrectomy	↓ BUN, s-creat, KiHPCh, systolic blood pressure, MDA; ↑ CrCl	[54]
*Hydrangea paniculata* stem water extract rich in coumarin glycosides (metabolized to umbelliferone and esculetin), 15, 30, and 45 mg/kg	(NP) (V) male Wistar rats, STZ	↓ BUN, s-creat, albuminuria, fibronectin, collagen 4, KiHPCh, ROS production, p-Smad2/3; ↑ CrCl, Nrf2	[59]
*Hypericum perforatum*, 50, 100, and 200 mg/kg for 8 weeks	(NP) (V) rats, nicotinamide + STZ	↓ s-glu, s-urea, s-creat, albuminuria, NF-*κ*B, iNOS, COX-2, collagen 4, fibronectin, MDA, NO, TNF-*α*, IL-1*β*, ICAM-1, MCP-1, TGF-*β*, caspase-3, and cytochrome c; ↑ s-insulin, PPAR*γ*, GSH, SOD	[31]
*Paederia foetida* methanolic leaf, 250 and 500 mg/kg	(NP) (V) Wistar rats, alloxan	↓ s-glu, s-creat, BUN, bilirubin, AST, ALT, TG, TC, TBARS/MDA, IL-6, IL-1*β*, TNF-*α*, NF-*κ*B activation, KiHPCh; ↑ GFR, s-albumin, ↑ activity of enzymatic and non-enzymatic antioxidants	[78]
*Paeonia emodi* roots flavonoid alcohol and hydroalcohol extract, 100, 200, and 400 mg/kg for 45 days	(NP) (V) male Wistar rats, nicotinamide + STZ	↓ s-glu, HbA1c, s-uric, s-creat, BUN, TC, TG, LDL, VLDL, KW/BW, polyuria, albuminuria, u-creat, KiHPCh, TBARS, AGEs; ↑ BW, s-insulin, HDL-C, CrCl, GSH, SOD	[63]
*Paeonia lactiflora* root—total glucosides, 50, 100, and 200 mg/kg for 8 weeks	(NP) (V) male Munich-Wistar rats, STZ	↓ albuminuria, glomerular volume, tubulointerstitial injury (ALT), collagen 4, ICAM-1, IL-1, TNF-*α*, NF-*κ*Bp65, 3-NT, TGF-*β*1; ↑ nephrin	[120]
*Paeonia suffruticosa* root bark (cortex Moutan), paeonol (1, 10, and 100 *μ*M), paeoniflorin (2, 20, and 200 *μ*M) or pentagalloylglucose (1, 10, and 100 *μ*M) for 48 h	(T) HG-cultured mesangial cells	*In vitro*: paeoniflorin, pentagalloylglucose, and paeonol ↓ NOX; ↓ ROS, TGF-*β*1, and fibronectin.	[109]
*Panax notoginseng* saponins, 50 and 200 mg/kg for 30 days, i.p.	(NP) (V) male KK-Ay (KK/UPJ-Ay/J) mice (animal model for human type 2 DN)	↓ s-glu, BW, insulin resistance, TG, glomerular lesions; ↑ glucose tolerance	[35]
*Panax quinquefolium* (American ginseng), 100 mg/kg for 20 days	(NP) (V) male Wistar rats, STZ	↓ KW, polyphagia, polydipsia, polyuria, proteinuria, s-glu, glycosylated protein, N*ε*-(carboxymethyl)lysine (an AGE), RAGE; ↑ BW, CrCl	[83]
*Panax quinquefolium* (north American ginseng)—root alcoholic extract, 200 mg/kg for 2 or 4 months	(NP) (V) C57BL/6 mice, STZ (T1DM model); *db/db* (a strain of T2DM) mice	↑ BW (decreased in T1DM model); ↓ BW, plasma insulin levels, insulin resistance (increased in T2DM model); ↓ s-glu, HbA1c, albuminuria, s-creat, oxidative stress, HO-1, NF-*κ*B, mesangial expansion, ECM, fibronectin, collagen 4-*α*1, VEGF, endothelin-1, TGF-*β*1	[82]
*Physalis angulata*—methanol extract of whole plant, 500 mg/kg orally for 14 days	(NP) (V) male Wistar rats, alloxan	↓ s-glu, KW, fructosamine, HbA1c, MDA, s-creat, BUN; ↑ BW, SOD	[64]
*Pleurotus eryngii* polysaccharides, 600 and 300 mg/kg for 16 days	(NP) (V) male Kunming mice, STZ	↓ s-glu, BUN, s-creat, s-uric, TC, TG, VLDL-C, LDL-C, MDA, KiHPCh, KW/BW, s-albumin (*sic*); ↑ BW, GPx, SOD, CAT	[57]
*Prunella vulgaris* aqueous extract, 100 and 300 mg/kg for 8 weeks	(NP) (T, V) male SD rats, STZ; HG-cultured (25 mM) human mesangial cell	↓ TGF-*β*, Smad-2/4, CTGF, collagen 4, ICAM-1, MCP-1, NF-*κ*B, ROS production, s-glu, BUN, s-creat, glomerular ECM, GBM thickening; ↑ Smad-7	[84]
*Psoralea corylifolia* seed extract (PCS), 500 mg/kg for 8 weeks; isopsoralen and psoralen, major components of PCS	(NP) (T, V) male C57BL/6 mice; HG-cultured mesangial MES-13 cells	↓ CrCl, polyuria, microalbuminuria, mesangial expansion, collagen 4-*α*2, fibronectin, plasminogen activator inhibitor-1, TGF-*β*1, apoptosis marker genes (cleaved PARP and Bcl-2-associated death promoter (Bad)); ↑ survival markers: (p-Bad (ser112) and Bcl-2)	[115]
*Punica granatum*—flavonoid-rich fraction of, 50, 100, and 200 mg/kg for 28 days	(NP) (V) Wistar rats, STZ	↓ s-glu, s-glu increase at OGTT, TC, TG, LDL-C, VLDL-C, HbA1c, proteinuria, albuminuria, s-creat, BUN, KW/BW, polyphagia, polydipsia, polyuria, MDA, KiHPCh including GS; ↑ BW, HDL-C, s-protein, s-albumin, CrCl, GSH, SOD, CAT	[32]
*Ramulus mori* (*Morus alba*) polysaccharides, 600 mg/kg for 30 days	(NP) (V) male BALB/c mice, STZ	↓s-glu, s-glycosylated protein, TC, BUN, s-creat, proteinuria, IL-6, interferon-*γ*, TNF-*α*, IL-1, IL-1 receptor, KiHPCh, p-I*κ*B, NF-*κ*B, ↑ s-albumin	[77]
*Rheum ribes*, root hydroalcoholic extract, 75 and 150 mg/kg for 28 days	(NP) (V) female Wistar rats, alloxan	↓ s-glu, TC, TG, LDL-C, s-uric, s-urea, s-creat, KiHPCh; ↑ HDL-C, BW	[175]
*Salacia chinensis*, 1000 mg bid for 6 months	(NP) (H) stable diabetic CKD patients. Low-quality (J1, i.e., the randomization method not described, the trial was not double-blinded, no description of withdrawals and dropouts), low-powered trial	↓ s-creat, progression of CKD, endothelial dysfunction markers (homocysteine, IL-6); ↑ CrCl	[102]
*Spirogyra neglecta* extract, 0.25, 0.5, and 1 g/kg for 12 weeks	(V) male Wistar rats, high-fat diet + STZ	↓ s-glu, TG, insulin resistance, KiHPCh, MDA, GPx, NF-*κ*B; ↑ insulin-stimulated rOat3, anion uptake	[176]
*Terminalia chebula*—chloroform extract of seed powder, 100, 200, and 300 mg/kg for 8 weeks	(NP) (V) SD rats, STZ	↓ s-glu; renoprotective	[33]
*Vigna angularis* (azuki bean), 10 or 40 mg/kg/d for 4 weeks	(V) male Wistar rats, STZ	↑ glutathione; ↓ HO-1, p47phox; ↑ autophagy (↑ LC3B-II, ↓ p62/sequestosome 1)	[146]

↓: decreased/prevented the increase (of activity (for enzymes)/level (for biochemical parameters))/inhibited/inactivated/blocked/suppressed/downregulated (about pathways, enzymes, receptors, cytokines, etc.); ↑: increased/prevented the decrease (of activity (for enzymes)/level (for biochemical parameters))/stimulated/activated/upregulated; →: leading to/inducing; 3-NT: 3-nitrotyrosine; AGEs: advanced glycation end-products; ALP: alkaline phosphatase; ALT: alanine aminotransferase/transaminase; AMPK: 5′ adenosine monophosphate-activated protein kinase; AST: aspartate aminotransferase/transaminase; Bax: Bcl-2-associated X protein; Bcl-2: B-cell lymphoma 2 protein; BUN: blood urea nitrogen; BW: body weight; CAT: catalase; Cd: cadmium (generally given as cadmium chloride CdCl2); COX-2: cyclooxygenase 2; CrCl: creatinine clearance; CTGF: connective tissue growth factor; DN: diabetic nephropathy; ECM: extracellular matrix; EMT: epithelial-mesenchymal transdifferentiation; eGFR: estimated glomerular filtration rate; eNOS: endothelial NO synthase; eNOS−/−: endothelial nitric oxide synthase-null; EP4: E prostanoid receptor 4; ER: endoplasmic reticulum; FFA: free fatty acids; GBM: glomerular basement membrane; GFR: glomerular filtration rate; GPx: glutathione peroxidase; GRed: glutathione reductase; GS: glomerulosclerosis; GSH: reduced glutathione; GST: glutathione S-transferase; HbA1c: glycated hemoglobin; HDL: high-density lipoprotein; HDL-C: HDL-cholesterol; HG: high glucose, i.e., glucose 30 mM if not otherwise specified (the physiological concentration being 5 mM); HIF-1*α:* hypoxia-inducible factor 1*α*; HO-1: heme oxygenase-1; ICAM: intercellular adhesion molecule; IL: interleukin; iNOS: inducible NO synthase; I*κ*B*α*: inhibitor of NF-*κ*B; JNK: c-Jun N-terminal kinase; KiHPCh: kidney histopathological changes; KIM-1: kidney injury molecule-1; KW: kidney weight; KW/BW: (kidney weight)/(body weight) = kidney index; LC3B-II: light chain 3B II; LDL: low-density lipoprotein; LDL-C: LDL-cholesterol; LPO: lipid peroxidation; MAPK: mitogen-activated protein kinase; MC: mesangial cells; MCP: monocyte chemoattractant/chemotactic protein; MDA: malondialdehyde; miR: microRNA; miR-141-3p: a member of the microRNA- (miR-) 200 family; MMP: matrix metalloproteinase; MPO: myeloperoxidase; NADPH: reduced nicotinamide-adenine dinucleotide phosphate; NF-*κ*B: nuclear factor kappa-B (nuclear factor kappa-light-chain-enhancer of activated B cells); NLRP3: nucleotide binding and oligomerization domain-like receptor family pyrin domain-containing 3; NO: nitric oxide; NOX: NADPH oxidase; Nrf2: nuclear factor erythroid-derived 2; OGTT: oral glucose tolerance test; p-: phospho-/phosphorylated; PARP: poly (ADP-ribose) polymerase; PI3K: phosphatidylinositol 3-kinase; PKC: proteinkinase C; PL: phospholipids; PPAR: peroxisome proliferator-activated receptor; PTEN: phosphatase and tensin homolog; RAGE: receptor for advanced glycation end-products; RAS: renin-angiotensin system; ROS: reactive oxygen species; s-: serum level of; s-creat: serum level of creatinine; SD: Sprague Dawley, a strain of rats; SERCA2b: sarcoendoplasmic reticulum Ca^2+^ ATPase 2b; s-glu: serum/plasma/blood level of glucose; SIRT1: sirtuin1; Smad proteins: signal transducers for receptors of TGF-*β* superfamily; SOD: superoxide dismutase; SREBP: sterol regulatory element-binding protein; STZ: streptozotocin; s-urea: serum level of urea; s-uric: serum level of uric acid; T1DM/T2DM: type 1/2 diabetes mellitus; TBARS: thiobarbituric acid reactive substances; TC: total cholesterol; TG: triglyceride/triacylglycerol; TGF-*β*: transforming/tumor growth factor *β*; TIMP: tissue inhibitor of MMPs; TNF-*α*: tumor necrosis factor-*α*; u-: urine level of; u-creat: urine level of creatinine; VEGF: vascular endothelial growth factor.

**Table 2:** Phytoagents active in diabetic nephropathy classified according to their mechanism of action. The marker (H) was used to signal human studies.

Aggressive factors	
Glucose metabolism	
Serum glucose level	(-)-Epicatechin 3-O-gallate and (-)-epigallocatechin 3-O-gallate [81], (-)-epigallocatechin-3-gallate [155], 20(S)-ginsenoside Rg(3) [169], *Allium sativum* [28], *Anogeissus acuminata* [56], *Artemisia campestris* [55], *Artemisia sieberi* [29], *Bacopa monnieri* [30], berberine [85] [117] [118] [121], *Boerhaavia diffusa* [172], *Chlorella pyrenoidosa* [173], *Cladophora glomerata* [34], *Coccinia indica* [111], coconut water [61], *Cordyceps militaris* [114], *Cornus officinalis* [47], curcumin [79], dihydroquercetin [48], *Dioscorea bulbifera* [96], diosgenin [62], ellagic acid [37], emodin§ [94], *Flammulina velutipes* [53], *Fragaria × ananassa* [89], Huangqi decoction [36], *Hypericum perforatum* [31], *Paederia foetida* [78], *Paeonia emodi* [63], palm oil and rice bran oil [50], *Panax notoginseng* [35], *Panax quinquefolium* [82], *Panax quinquefolium* [83], *Physalis angulata* [64], *Pleurotus eryngii* polysaccharides [57], *Prunella vulgaris* [84], *Punica granatum* [32], quercetin [49], *Ramulus mori* [77], *Rheum ribes* [175], secoisolariciresinol diglucoside [88], *Spirogyra neglecta* [176], Tangke decoction [116], *Terminalia chebula* [33]
Insulin resistance	*Cladophora glomerata* [34], ellagic acid [37], Huangqi decoction [36], *Panax notoginseng* [35], *Panax quinquefolium* [82], *Spirogyra neglecta* [176]

Oxidative/nitrosative stress	
Oxidative stress	(-)-Epigallocatechin-3-gallate [155], *Abelmoschus manihot* [156], *Allium sativum* [28], *Artemisia campestris* [55], berberine [95] (human), berberine [44], celastrol [170], cocoa enriched with polyphenols [110], *Cornus officinalis* [47], crocin [73], curcumin [41], dihydroquercetin [48], d-pinitol [2], ferulic acid [145], *Hydrangea paniculata* [59], moringa isothiocyanate [42], *Paeonia suffruticosa* [109], *Panax quinquefolium* [82], *Prunella vulgaris* [84], puerarin [76], puerarin [60], quercetin [49], silybin [43], *Vigna angularis* [146]
Lipid peroxidation (LPO)	(-)-Epicatechin 3-O-gallate and (-)-epigallocatechin 3-O-gallate [81], (+)-catechin [46], *Abelmoschus manihot* [156], *Allium sativum* [28], *Anogeissus acuminata* [56], *Artemisia campestris* [55], *Bacopa monnieri* [30], berberine [95] (human), *Boerhaavia diffusa* [172], breviscapine [51], breviscapine [52], *Cornus officinalis* [47], curcumin [108], curcumin [41], diosgenin [62], d-pinitol [2], ellagic acid [37], emodin [94], ferulic acid [145], *Flammulina velutipes* [53], *Fragaria × ananassa* [89], *Hibiscus sabdariffa* [54], *Hypericum perforatum* [31], isorhamnetin [87], *Paederia foetida* [78], *Paeonia emodi* [63], *Paeonia suffruticosa* [40], *Panax ginseng* [169], *Physalis angulata* [64], *Pleurotus eryngii* [57], *Punica granatum* [32], secoisolariciresinol diglucoside [88], silymarin [65] (human), *Spirogyra neglecta* [176]
Nitrosative stress	(-)-Epicatechin 3-O-gallate and (-)-epigallocatechin 3-O-gallate [81], *Abelmoschus manihot* [156], *Allium sativum* [28], *Artemisia campestris* [55], breviscapine [52], cocoa enriched with polyphenols [110], *Cornus officinalis* [47], curcumin [108], curcumin analogue (B06) [66], d-pinitol [2], *Hypericum perforatum* [31], moringa isothiocyanate [42], *Paeonia lactiflora* [120], *Paeonia suffruticosa* [40], puerarin [76], puerarin [60], secoisolariciresinol diglucoside [88], silybin [43], ferulic acid [145]

Antioxidant protection	
Antioxidant capacity	Berberine [95] (human), *Paederia foetida* [78]
Catalase (CAT)	(-)-Epicatechin 3-O-gallate and (-)-epigallocatechin 3-O-gallate [81], *Anogeissus acuminata* [56], *Artemisia campestris* [55], *Boerhaavia diffusa* [172], breviscapine [51], *Coccinia indica* [111], diosgenin [62], d-pinitol [2], ellagic acid [37], *Flammulina velutipes* [53], *Fragaria × ananassa* [89], *Paeonia suffruticosa* [40], *Pleurotus eryngii* [57], *Punica granatum* [32], ferulic acid [145]
Free thiols	(+)-Catechin [46]
Glutathione peroxidase (GPx)	(-)-Epicatechin 3-O-gallate and (-)-epigallocatechin 3-O-gallate [81], *Artemisia campestris* [55], *Boerhaavia diffusa* [172], breviscapine [51], *Coccinia indica* [111], coconut water [61], curcumin [108], d-pinitol [2], ellagic acid [37], *Flammulina velutipes* [53], *Paeonia suffruticosa* [40], *Pleurotus eryngii* [57], *Spirogyra neglecta* [176]
Glutathione reductase (GRed)	Coccinia indica [111], d-pinitol [2]
Reduced glutathione (GSH)	(-)-Epicatechin 3-O-gallate and (-)-epigallocatechin 3-O-gallate [81], *Anogeissus acuminata* [56], *Artemisia campestris* [55], *Bacopa monnieri* [30], *Boerhaavia diffusa* [172], *Cornus officinalis* [47], diosgenin [62], d-pinitol [2], ellagic acid [37], Hypericum perforatum [31], *Paeonia emodi* [63], *Punica granatum* [32], secoisolariciresinol diglucoside [88], berberine [44], ferulic acid [145]
Glutathione S-transferase (GST)	*Coccinia indica* [111], d-pinitol [2]
Superoxide dismutase (SOD)	(-)-Epicatechin 3-O-gallate and (-)-epigallocatechin 3-O-gallate [81], *Abelmoschus manihot* [156], *Artemisia campestris* [55], *Bacopa monnieri* [30], berberine [95] (human), Boerhaavia diffusa [172], breviscapine [51], crocin [73], curcumin [108], curcumin [41], diosgenin [62], d-pinitol [2], ellagic acid [37], emodin [94], *Flammulina velutipes* [53], *Hypericum perforatum* [31], isorhamnetin [87], *Paeonia emodi* [63], *Paeonia suffruticosa* [40], *Physalis angulata* [64], *Pleurotus eryngii* [57], *Punica granatum* [32], secoisolariciresinol diglucoside [88], berberine [44], ferulic acid [145]

Advanced glycation end-products (AGEs)	
AGEs	(-)-Epicatechin 3-O-gallate and (-)-epigallocatechin 3-O-gallate [81], *Bacopa monnieri* [30], berberine [118], coconut water [61], *Cordyceps militaris* [114], *Cornus officinalis* [47], curcumin [41], diosgenin [62], d-pinitol [2], *Paeonia emodi* [63], *Paeonia suffruticosa* [40], *Panax quinquefolium* [83], ferulic acid [145]
Receptor for AGEs (RAGE)	Berberine [118], *Panax quinquefolium* [83]

Autophagy	
AMPK	Astragaloside IV [103], berberine [136]
AMPK/mTOR	*Cyclocarya paliurus*—triterpenic acids-enriched fraction [142], astragaloside IV [103], berberine [136]
AMPK/mTOR/ULK1	Mangiferin [139]
HO-1	Celastrol [170]
Hypoxic stress: SIRT-1, Foxo3/Bnip3/Hif1a	Resveratrol [144]
MAPKs (p38/, JNK, ERK 1/2), NF-*κ*B	Ferulic acid [145]
miR-141-3p/PTEN/Akt/mTOR	Triptolide [141]
miR-383-5p	Resveratrol [168]
miRNA-18a-5p	Resveratrol [133]
Oxidative stress (HO-1, p47phox)	*Vigna angularis* [146]
PI3K/AKT	Curcumin [132], berberine [44]
Pim1/p21/mTOR	Hispidulin [137]
Promyelocytic leukemia zinc finger protein	Tangshen Formula [123]
SIRT-1	*Abelmoschus manihot* [143]
SIRT1/NF-*κ*B	astragaloside IV [131]
*β*-Arrestin-1	Tripterygium glycosides [138]

Pathways	
Akt	(-)-Epigallocatechin-3-gallate [155], *Abelmoschus manihot* [156], emodin [94]
Angiotensin II (AT2)	(-)-Epigallocatechin-3-gallate [155]
Inducible nitric oxide synthase (iNOS)	(-)-Epicatechin 3-O-gallate and (-)-epigallocatechin 3-O-gallate [81], *Cornus officinalis* [47], curcumin [108], curcumin analogue (B06) [66], *Hypericum perforatum* [31], moringa isothiocyanate [42], secoisolariciresinol diglucoside [88]
Cyclooxygenase 2 (COX-2)	(-)-Epicatechin 3-O-gallate and (-)-epigallocatechin 3-O-gallate [81], *Cornus officinalis* [47], curcumin analogue (B06) [66], *Hypericum perforatum* [31]
Endoplasmic reticulum stress (ER stress)	*Abelmoschus manihot* [104], astragaloside IV [15], astragaloside IV [103]
c-Jun NH2-terminal kinase (JNK)	*Abelmoschus manihot* [104]
Extracellular regulated protein kinases (ERK1/2)	(-)-Epigallocatechin-3-gallate [155]
Glycogen synthase kinase 3*β* (GSK-3*β*)	Emodin [94]
Nuclear factor *κ*B (NF-*κ*B)	(-)-Epicatechin 3-O-gallate and (-)-epigallocatechin 3-O-gallate [81], berberine [85], *Coreopsis tinctoria* [72], *Cornus officinalis* [47], crocin [73], curcumin [79], curcumin analogue (B06) [66], d-pinitol [2], ellagic acid [37], *Fragaria × ananassa* [89], *Hypericum perforatum* [31], isorhamnetin [87], *Morus alba* [77], *Paederia foetida* [78], *Paeonia lactiflora* [120], *Panax quinquefolium* [82], *Prunella vulgaris* [84], puerarin [76], *Zea mays* anthocyanins [70], secoisolariciresinol diglucoside [88], Shen-Yan-Fang-Shuai Formula [80], *Spirogyra neglecta* [176], astragaloside IV [131], *Abelmoschus manihot* [143], ferulic acid [145]
Nucleotide binding and oligomerization domain-like receptor family pyrin domain-containing 3 (NLRP3) inflammasome	Dihydroquercetin [48]
Smad (TGF-*β*/Smad signaling)	Berberine [85], cocoa enriched with polyphenols [110], *Coreopsis tinctoria* [72], *Hydrangea paniculata* [59], *Prunella vulgaris* [84], *Zea mays* anthocyanins [70], Tangke decoction [116]
Sterol regulatory element-binding protein (SREBP)	*Cornus officinalis* [47], curcumin [108], *Fragaria × ananassa* [89], myricetin [157]
Transforming/tumor growth factor *β* (TGF-*β*)	(-)-Epicatechin 3-O-gallate and (-)-epigallocatechin 3-O-gallate [81], *Abelmoschus manihot* [104], *Abelmoschus manihot* [156], berberine [121], berberine [85], berberine [117], berberine [118], breviscapine [51], breviscapine [52], cocoa enriched with polyphenols [110], *Cordyceps militaris* [114], *Coreopsis tinctoria* [72], curcumin [108], curcumin [79], curcumin analogue (B06) [66], *Dioscorea bulbifera* [96] (human), ellagic acid [37], *Hypericum perforatum* [31], isorhamnetin [87], moringa isothiocyanate [42], myricetin [157], *Paeonia lactiflora* [120], *Paeonia suffruticosa* [109], *Panax quinquefolium* [82], *Prunella vulgaris* [84], *Psoralea corylifolia* [115], *Zea mays* anthocyanins [70], Qidan Dihuang [119], rhein [113], Shen-Yan-Fang-Shuai Formula [80], Tangke decoction [116]
p38-mitogen-activated protein kinase (p38MAPK)	(-)-Epigallocatechin-3-gallate [155], *Abelmoschus manihot* [156], Danhong [154], emodin [153]
Phosphatidylinositol 3-kinase (PI3K)	(-)-Epigallocatechin-3-gallate [155], Huangqi decoction [36]
Proteinkinase C (PKC)	Berberine [118], breviscapine [51], breviscapine [52], *Cladophora glomerata* [34]
JNK (JNK-NF-*κ*B signaling)	Astragaloside IV [15], curcumin analogue (B06) [66]
Insulin receptor (IR) and insulin receptor substrate (IRS)	Huangqi decoction [36]
Glucose transporter 1 and 4 (GLUT1, GLUT4)	Huangqi decoction [36]
G protein-coupled receptor kinase (GRK)2, GRK3, GRK6	Berberine [121]
PGE2/EP1/G*α*q/Ca^2+^	Berberine [122]
cAMP response element-binding protein (CREB)	Emodin [153]
Cyclic adenosine monophosphate (cAMP)	Berberine [158], berberine [121], emodin [153]

Mediators	
Matrix metalloproteinase (MMP)	
MMP-1	*Zea mays* anthocyanins [70]
MMP-9 and MMP-9/TIMP-1 ratio	Berberine [117], puerarin [60], rhein [127]
Tissue inhibitor of MMPs- (TIMP-) 2 and MMP-2/TIMP-2 ratio	Berberine [117], *Zea mays* anthocyanins [70],

Cytokines	
IL-1 (interleukin-1)	*Abelmoschus manihot* [104], berberine [85], crocin [73], curcumin [79], curcumin analogue (B06) [66], dihydroquercetin [48], d-pinitol [2], ellagic acid [37], *Hypericum perforatum* [31], isorhamnetin [87], moringa isothiocyanate [42], *Morus alba* [77], *Paederia foetida* [78], *Paeonia lactiflora* [120], celastrol [170], resveratrol [144], ferulic acid [145]
IL-1 receptor (IL-1R)	*Morus alba* [77]
IL-10	Crocin [73], resveratrol [144]
IL-12	Curcumin analogue (B06) [66]
IL-2	*Abelmoschus manihot* [104]
IL-6	*Abelmoschus manihot* [104], curcumin analogue (B06) [66], *Dioscorea bulbifera* [96] (human), d-pinitol [2], ellagic acid [37], emodin [94], *Fragaria × ananassa* [89], isorhamnetin [87], moringa isothiocyanate [42], *Morus alba* [77], *Paederia foetida* [78], *Paeonia suffruticosa* [40], *Salacia chinensis* [102] (human), celastrol [170], resveratrol [144], *Abelmoschus manihot* [143], ferulic acid [145]
IL-8	Crocin [73]
Interferon-*γ*	*Morus alba* [77]
Tumor necrosis factor-*α* (TNF-*α*)	*Abelmoschus manihot* [104], *Abelmoschus manihot* [156], *Allium sativum* [28], berberine [85], crocin [73], curcumin [79], curcumin analogue (B06) [66], d-pinitol [2], ellagic acid [37], emodin [94], *Fragaria × ananassa* [89], *Hypericum perforatum* [31], isorhamnetin [87], *Morus alba* [77], *Paederia foetida* [78], *Paeonia lactiflora* [120], secoisolariciresinol diglucoside [88], Shen-Yan-Fang-Shuai Formula [80], silymarin [65] (human), astragaloside IV [103], celastrol [170], resveratrol [144], *Abelmoschus manihot* [143], ferulic acid [145]

Chemokines	
Monocyte chemoattractant/chemotactic protein-1 (MCP-1)	berberine [85], breviscapine [51], *Coreopsis tinctoria* [72], curcumin [79], curcumin analogue (B06) [66], *Hypericum perforatum* [31], moringa isothiocyanate [42], *Paeonia suffruticosa* [40], *Prunella vulgaris* [84], *Zea mays* anthocyanins [70], Shen-Yan-Fang-Shuai Formula [80], astragaloside IV [103], ferulic acid [145]

Adhesion molecules	
Intercellular adhesion molecule-1 (ICAM-1)	Breviscapine [51], curcumin [79], emodin [94], *Hypericum perforatum* [31], isorhamnetin [87], *Paeonia lactiflora* [120], *Prunella vulgaris* [84], *Zea mays* anthocyanins [70], ferulic acid [145]
Vascular cell adhesion molecule-1 (VCAM-1)	Berberine [95], ferulic acid [145]

Growth factors	
Vascular endothelial growth factor (VEGF)	Myricetin [157], *Panax quinquefolium* [82]
Connective tissue growth factor (CTGF)	Emodin [153], *Prunella vulgaris* [84], *Zea mays* anthocyanins [70]

Cellular infiltration	
Macrophage infiltration	Breviscapine [51], curcumin [79], curcumin analogue (B06) [66]
Macrophage migration	*Paeonia suffruticosa* [40]
Myeloperoxidase (MPO)	(-)-Epicatechin 3-O-gallate and (-)-epigallocatechin 3-O-gallate [81], diosgenin [62]

Diabetic nephropathy (DN)	
Apoptosis	
Apoptosis	Astilbin [140], astragaloside IV [15], curcumin [41], *Psoralea corylifolia* [115], quercetin [49], secoisolariciresinol diglucoside [88], silybin [43], berberine [45], berberine [44], berberine [136], tripterygium glycosides [138], celastrol [170], curcumin [132], resveratrol [133], astragaloside IV [103], *Cyclocarya paliurus*—triterpenic acids-enriched fraction [142], ferulic acid [145]
HIF-1*α*/PI3K/Akt	Berberine [45]
Bcl-2-associated X protein (BAX)	Astilbin [140], emodin [94], berberine [45], berberine [44]
B-cell lymphoma 2 protein (Bcl-2)	Astilbin [140], emodin [94], *Psoralea corylifolia* [115], secoisolariciresinol diglucoside [88]
Bcl-2-associated death promoter (Bad)	*Psoralea corylifolia* [115]
Caspase-9	Berberine [45], berberine [44], ferulic acid [145]
Caspase-12	Astragaloside IV [103]
Caspase-3	Astilbin [140], astragaloside IV [15], emodin [94], *Hypericum perforatum* [31], berberine [45], berberine [44], curcumin [132], resveratrol [133], resveratrol [168], astragaloside IV [103], *Cyclocarya paliurus*—triterpenic acids-enriched fraction [142], ferulic acid [145]
Caspase-1	Dihydroquercetin [48]
Poly (ADP-ribose) polymerase (PARP)	*Psoralea corylifolia* [115]
Survivin (antiapoptotic marker)	Secoisolariciresinol diglucoside [88]
C/EBP homologous protein (CHOP)	Astragaloside IV [15] [103]

Glomerulosclerosis (GS), fibrosis, extracellular matrix (ECM) expansion	
Collagen 1	Berberine [85]
Collagen 3	Tangshen Formula [123]
Collagen 4	*Abelmoschus manihot* [104], berberine [121], berberine [85], berberine [117], cocoa enriched with polyphenols [110], *Cordyceps militaris* [114], *Coreopsis tinctoria* [72], dihydroquercetin [48], *Hydrangea paniculata* [59], *Hypericum perforatum* [31], myricetin [157], *Paeonia lactiflora* [120], *Panax quinquefolium* [82], *Prunella vulgaris* [84], *Psoralea corylifolia* [115], *Zea mays* anthocyanins [70], Shen-Yan-Fang-Shuai Formula [80], triptolide [133], astragaloside IV [131]
Fibronectin	(-)-Epicatechin 3-O-gallate and (-)-epigallocatechin 3-O-gallate [81], (+)-catechin [46], berberine [85], berberine [117], *Coccinia indica* [111], cocoa enriched with polyphenols [110], *Coreopsis tinctoria* [72], dihydroquercetin [48], ellagic acid [37], emodin [153], *Hydrangea paniculata* [59], *Hypericum perforatum* [31], myricetin [157], *Paeonia suffruticosa* [109], *Panax quinquefolium* [82], *Psoralea corylifolia* [115], rhein [113], Shen-Yan-Fang-Shuai Formula [80], triptolide [133], astragaloside IV [131]
Laminin	*Coccinia indica* [111]

Endothelial dysfunction	
Endothelial dysfunction	*Salacia chinensis* [102] (H)
Endothelin-1	(+)-Catechin [46], *Panax quinquefolium* [82]
Homocysteine	*Salacia chinensis* [102] (H)

Podocytes, foot processes, slit diaphragms	
Podocyte apoptosis	Silybin [43], celastrol [170], resveratrol [168]
Podocyte foot processes effacement	Curcumin [130], puerarin [60]
Podocytes CD2-associated protein (markers of slit diaphragms reduced by HG)	Crocin [73]
Podocyte slit diaphragm proteins (nephrin, podocin, and synaptopodin)	Puerarin [60]
Podocin	Crocin [73], puerarin [60], hispidulin [137], berberine [136], tripterygium glycosides [138], astragaloside IV [103]
Nephrin	Crocin [73], *Paeonia lactiflora* [120], puerarin [60], mangiferin [136], hispidulin [137], berberine [136], tripterygium glycosides [138], celastrol [170], resveratrol [168], astragaloside IV [103]
Synaptopodin	Curcumin [108]
Desmin	Curcumin [108]

Epithelial-mesenchymal transdifferentiation (EMT)	
Caveolin-1, *β*-catenin	Curcumin [130]
Integrin-linked kinase	Rhein [127]
Vimentin	Curcumin [108]

Protectors	
Akt	Astilbin [140], Huangqi decoction [36]
Nuclear factor erythroid-derived 2 (Nrf2)	*Hydrangea paniculata* [59], moringa isothiocyanate [42], berberine [44]
NAD(P)H:Quinone oxidoreductase 1 (NOX1)	Moringa isothiocyanate [42]
Glutamate cysteine ligase catalytic subunit (GCLC)	Moringa isothiocyanate [42]
5′ adenosine monophosphate-activated protein kinase (AMPK)	Cocoa enriched with polyphenols [110], *Coreopsis tinctoria* [72]
Sirtuin 1 (SIRT1)	Puerarin [76]
Peroxisome proliferator-activated receptor-*α* (PPAR-*α*)	*Abelmoschus manihot* [104], myricetin [157]
Peroxisome proliferator-activated receptor-*γ* (PPAR-*γ*)	*Abelmoschus manihot* [104], Danhong [154], emodin [153], *Fragaria × ananassa* [89], *Hypericum perforatum* [31]
E prostanoid receptor 4/protein G*α*s/adenylate cyclase/cyclic adenosine monophosphate (EP4/G*α*s/AC/cAMP)	Berberine [158]

## Abbreviations

AC: Adenylyl/adenylate cyclase
AGEs: Advanced glycation end-products
AMPK: 5′ adenosine monophosphate-activated protein kinase
cAMP: Cyclic adenosine monophosphate
COX-2: Cyclooxygenase 2
DN: Diabetic nephropathy
ECM: Extracellular matrix
eGFR: Estimated glomerular filtration rate
EMT: Epithelial-mesenchymal transdifferentiation
EP4: E prostanoid receptor 4
G*α*s: G*α*s protein
ERK: Extracellular signal-regulated kinase
ER: Endoplasmic reticulum
Gs: Gs protein
GSK: Glycogen synthase kinase
HG: High glucose
ICAM: Intercellular adhesion molecule
IL: Interleukin
ILK: Integrin-linked kinase
J1*/*J2*/*J3*/*J4*/*J5: Score of 1*/*2*/*3*/*4*/*5 on the Jadad scoring system for the assessment of clinical trial quality
JNK: c-Jun N-terminal kinase
LPO: Lipid peroxidation
MAPK: Mitogen-activated protein kinase
MCP: Monocyte chemoattractant/chemotactic protein
MMP: Matrix metalloproteinase
mTOR: Mammalian/mechanistic target of rapamycin
NADPH: Reduced nicotinamide-adenine dinucleotide phosphate
NF-*κ*B: Nuclear factor kappa-B (nuclear factor kappa-light-chain-enhancer of activated B cells)
NO: Nitric oxide
NOX4: NADPH oxidase 4
Nrf2: Nuclear factor erythroid-derived 2
p-: Phospho-/phosphorylated
PI3K: Phosphatidylinositol 3-kinase
PKC: Proteinkinase C
PPAR: Peroxisome proliferator-activated receptor
RAGE: Receptor for AGEs
RAS: Renin-angiotensin system
ROS: Reactive oxygen species
SIRT1: Sirtuin1
Smad proteins: Signal transducers for receptors of TGF-*β* superfamily, critically important for regulating cell development and growth
SREBP: Sterol regulatory element-binding protein
TGF-*β*1: Transforming/tumor growth factor-*β*1
TIMP: Tissue inhibitor of MMPs
TNF-*α*: Tumor necrosis factor-*α*.
